# Crystal Growth from Anhydrous HF Solutions of M^2+^ (M = Ca, Sr, Ba) and [AuF_6_]^−^, Not Only Simple M(AuF_6_)_2_ Salts

**DOI:** 10.1021/acs.inorgchem.2c01675

**Published:** 2022-06-30

**Authors:** Zoran Mazej, Evgeny Goreshnik

**Affiliations:** Department of Inorganic Chemistry and Technology, Jožef Stefan Institute, Jamova cesta 39, SI-1000 Ljubljana, Slovenia

## Abstract

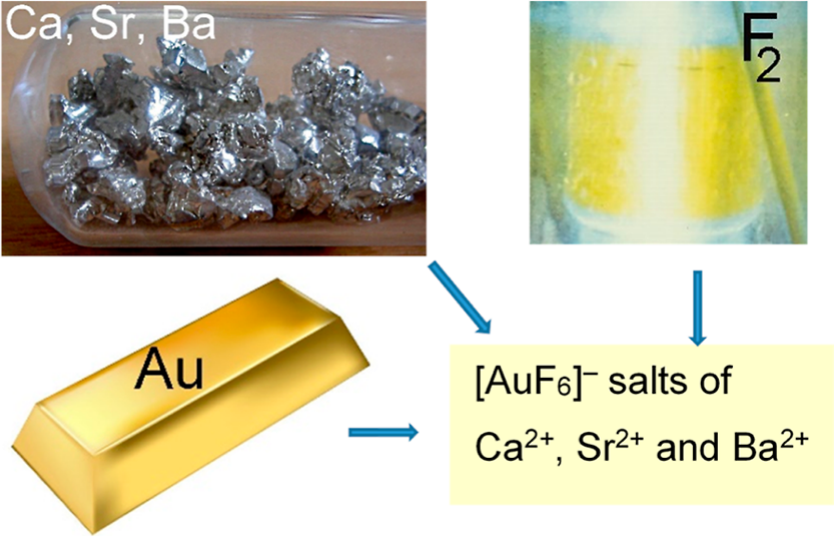

Crystal growth from anhydrous HF solutions of M^2+^ (M = Ca, Sr, Ba) and [AuF_6_]^−^ (molar ratio 1:2) gave [Ca(HF)_2_](AuF_6_)_2_, [Sr(HF)](AuF_6_)_2_, and Ba[Ba(HF)]_6_(AuF_6_)_14_. [Ca(HF)_2_](AuF_6_)_2_ exhibits a layered structure in which [Ca(HF)_2_]^2+^ cations are connected by AuF_6_ units, while the crystal structure of Ba[Ba(HF)]_6_(AuF_6_)_14_ exhibits a complex three-dimensional (3-D) network consisting of Ba^2+^ and [Ba(HF)_2_]^2+^ cations bridged by AuF_6_ groups. These results indicate that the previously reported M(AuF_6_)_2_ (M = Ca, Sr, Ba) compounds, prepared in the anhydrous HF, do not in fact correspond to this chemical formula. When the initial M^2+^/[AuF_6_]^−^ ratio was 1:1, single crystals of [M(HF)](H_3_F_4_)(AuF_6_) were grown for M = Sr. The crystal structure consists of a 3-D framework formed by [Sr(HF)]^2+^ cations associated with [AuF_6_]^−^ and [H_3_F_4_]^−^ anions. The latter exhibits a Z-shaped conformation, which has not been observed before. Single crystals of M(BF_4_)(AuF_6_) (M = Sr, Ba) were grown when a small amount of BF_3_ was present during crystallization. Sr(BF_4_)(AuF_6_) crystallizes in two modifications. A high-temperature α-phase (293 K) crystallized in an orthorhombic unit cell, and a low-temperature β-phase (150 K) crystallized in a monoclinic unit cell. For Ba(BF_4_)(AuF_6_), only an orthorhombic modification was observed in the range 80–230 K. An attempt to grow crystals of Ca(BF_4_)(AuF_6_) failed. Instead, crystals of [Ca(HF)](BF_4_)_2_ were grown and the crystal structure was determined. During prolonged crystallization of [AuF]_6_^–^ salts, moisture can penetrate through the walls of the crystallization vessel. This can lead to partial reduction of Au(V) to A(III) and the formation of [AuF_4_]^−^ byproducts, as shown by the single-crystal growth of [Ba(HF)]_4_(AuF_4_)(AuF_6_)_7_. Its crystal structure consists of [Ba(HF)]^2+^ cations connected by AuF_6_ octahedra and square-planar AuF_4_ units. The crystal structure of the minor product [O_2_]_2_[Sr(HF)]_5_[AuF_6_]_12_·HF was also determined.

## Abstract 2

Crystal growth from anhydrous HF solutions of M^2+^ (M = Ca, Sr, Ba) and [AuF_6_]^−^ (molar ratio 1:2) gave [Ca(HF)_2_](AuF_6_)_2_ and Ba[Ba(HF)]_6_(AuF_6_)_14_. These results indicate that the previously reported M(AuF_6_)_2_ (M = Ca, Sr, Ba) compounds do not correspond to this chemical formula. When the initial Sr^2+^/[AuF_6_]^−^ ratio was 1:1, crystals of [Sr(HF)](H_3_F_4_)(AuF_6_) were grown. Single crystals of M(BF_4_)(AuF_6_) (M = Sr, Ba) were obtained when a small amount of BF_3_ was present during crystallization. The crystal structures of [Ba(HF)]_4_(AuF_4_)(AuF_6_)_7_, [O_2_]_2_[Sr(HF)]_5_[AuF_6_]_12_·HF, and Ca(BF_4_)(AuF_6_) byproducts were also determined.

## Introduction

Although gold can occur in various oxidation states from −1 to +5, its chemistry is dominated by oxidation states (I) and (III).^1,2^ For most people, the only interesting oxidation state of gold is Au^0^, where we naturally think of the metal gold. It has attracted people for thousands of years because of its lustrous appearance. One of the goals of alchemists was to turn some other metals into gold. However, it is also interesting to scientists because of many other aspects. It is a unique element because it has very large relativistic effects, greater than any other element with *Z* < 100.^3^ This has a major effect on the chemical properties of gold and its compounds.^4,5^ An example is the stability of alkali or alkaline-earth metal aurides (RbAu, CsAu, CsAu·NH_3_, and BaAu_2_)^5,6^ and other compounds with Au^–^ anions such as the exotic oxides M_3_AuO (M = K, Rb, Cs).^7^

Gold(I) has been used extensively in various organic gold complexes that have been shown to have physiological therapeutic value, in contrast to gold(III), whose organic complexes have been shown to be toxic.^8^ With the exception of AuF, all other gold(I) halides are known in the condensed state.^1,9^ The molecular AuF was characterized in the gas phase.^10^ In the solid state, it was stabilized by an N-heterocyclic carbene ligand.^11^ In F_3_As–Au^+^SbF_6_^–^ there are strong cationic–anionic interactions through a fluorine atom.^12^ Therefore, the description as F_3_As–Au–F···SbF_5_ is also possible. Reports of true gold(II) compounds are sparse.^13−16^ A number of apparent gold(II) complexes are actually mixed gold(I)–gold(III) compounds; AuCl_2_ is Au_2_Au_2_^III^Cl_8_.^1^ Gold(III) is probably the most prominent oxidation state. AuF_3_, AuCl_3_, and AuBr_3_ are known in the condensed state.^1,17^ There are a large number of Au(III) salts containing the square-planar AuF_4_^–^ anion.^18−21^ Of the gold oxides, only the brown Au_2_O_3_ is of some importance. This Au(III) oxide is thermodynamically unstable, but its decay is kinetically inhibited up to a temperature of about 150–170 °C.^7^ On the other hand, there are a large number of organogold(III) compounds.^1,8,22,23^ The experimental preparation of gold(IV) compounds is still beyond our capabilities. The published structure of bis-benzene-1,2-dithiolato-Au(IV)^24^ later proved to be a compound of Au(III).^25^ A somewhat more recent report of Au(IV) compounds with similar ligands^26^ is therefore still awaiting confirmation, and the preparation of AuF_4_ is still a domain of theoretical chemistry.^27,28^ Gold(V) has only been observed in AuF_5_ and various [AuF_6_]^−^ salts.^1,2^ It is the highest known oxidation state of gold.^29^ Since the first report of [AuF_6_]^−^ salts ([Xe_2_F_11_][AuF_6_] and CsAuF_6_) in 1972,^30^ fewer than 40 other examples have become known (Table S1). [Xe_2_F_11_][AuF_6_] was synthesized by fluorination of AuF_3_ in the presence of excess XeF_6_ at elevated temperatures.^29^ Room-temperature syntheses require the use of strong oxidizing species such as KrF_2_,^31^ UV-irradiated F_2_,^32^ or O_2_F radicals.^33^ The reactions are usually carried out in anhydrous HF (aHF) as the solvent. Besides the crystal structure of AuF_5_,^34^ the number of known crystal structures of other Au(V) compounds is very limited. Examples with nonmetallic cations include [AuF_6_]^−^ salts of [O_2_]^+^ (low and high-temperature forms),^35^ [KrF]^+^,^34^ and [Xe_2_F_11_]^+^.^36^ X-ray single-crystal structures have also been determined for some [AuF_6_]^−^ salts with M^+^ and M^+2^ metal cations. Well-known examples include the crystal structures of KAuF_6_,^37^ M(AuF_6_)_2_ (M = Cd,^36^ Hg^38^), Mg(HF)AuF_4_AuF_6_,^36^ and AgFAuF_6_.^37^

Reactions between KrF_2_ and mixtures of MF_2_ and metallic Au (molar ratio 1:2) in aHF (aHF) as the solvent were assumed to lead to M(AuF_6_)_2_ (M = Ca, Sr, Ba) salts.^39^ These reactions were later reexamined using two different approaches, that is, KrF_2_ and UV-irradiated F_2_ in aHF.^40,41^ From the powder X-ray diffraction data, the Ca-salt was found to crystallize in the tetragonal unit cell and Sr and Ba salts in the cubic system.^38,42^ In all cases, pure M(AuF_6_)_2_ salts were assumed to be isolated. However, in one work, it was already suspected that the Ba salt was not a true Ba(AuF_6_)_2_ compound.^39^ The next problem was that the Raman spectra of these salts from different sources only partially agreed.^38,39,41^ For this reason, we made a great effort to prepare the M(AuF_6_)_2_ crystals from the corresponding solutions. We also investigated the possibility of preparing MFAuF_6_ (M = Ca, Sr, Ba) and M(BF_4_)(AuF_6_) salts with mixed anions. Unintentionally, single crystals of [O_2_]_2_[Sr(HF)]_5_[AuF_6_]_12_·HF were grown and the crystal structure was determined. The results of these experiments are described in the present work.

## Results and Discussion

Crystals were grown from saturated solutions prepared in two similar ways. In the first method, “M(AuF_6_)_2_” salts were prepared by reactions between MF_2_/2AuF_3_ and KrF_2_ or UV-irradiated F_2_ in aHF.^39^ After yellow solutions were obtained, the volatile phases were pumped off. The solid products were recovered, characterized, and redissolved in aHF to obtain saturated solutions from which crystals were attempted to grow (Table S2). In the second method, MF_2_/2AuF_3_ mixtures were treated with UV-irradiated F_2_ in aHF until yellow solutions were obtained and crystallization was attempted without prior isolation (Table S3). In both cases, trace amounts of oxygen or moisture in the reaction mixture or in the crystal growth solutions were the main problem. If present during synthesis, O_2_AuF_6_^34^ can be easily formed as a byproduct. During prolonged crystallization, moisture can penetrate through the FEP reaction vessel walls.^43−46^ This can lead to partial reduction of Au(V) to Au(III) and formation of [AuF_4_]^−^ byproducts. For example, instead of the desired single crystals of Mg(AuF_6_)_2_, single crystals of [Mg(HF)](AuF_4_)(AuF_6_) were grown.^36^

**Figure 1:**
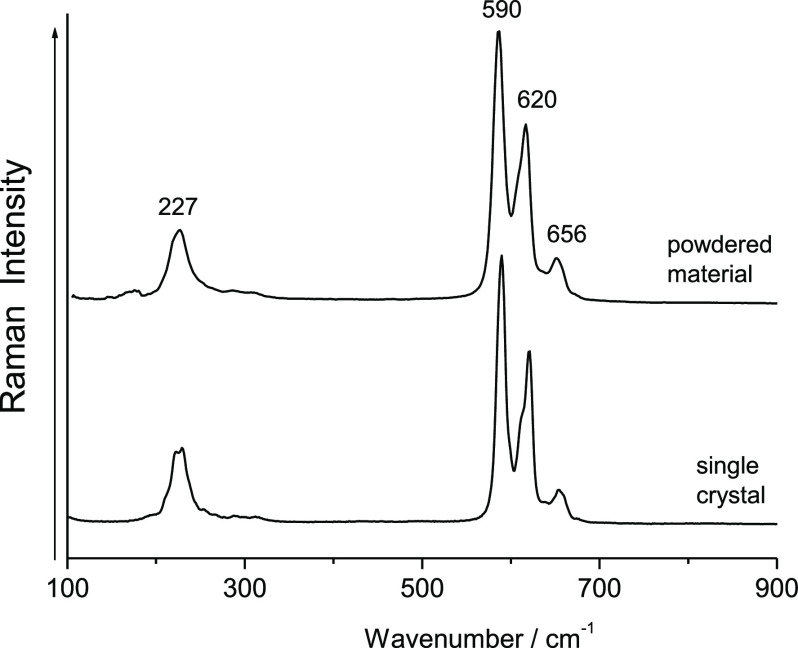
Raman spectrum of powdered [Ca(HF)_2_](AuF_6_)_2_^40^ and Raman spectrum of [Ca(HF)_2_](AuF_6_)_2_ recorded on single crystals checked using a diffractometer.

The major products grown from aHF solutions of Ca^2+^ and [AuF_6_]^−^ (molar ratio 1:2) were single crystals of [Ca(HF)_2_](AuF_6_)_2_. The Raman spectra, recorded on the single crystals (Figures 1 and S1), show similar features to those reported for Ca(AuF_6_)_2_.^38,39,41^

There are three possibilities: (a) both salts have very similar spectra; (b) the single crystals of [Ca(HF)_2_](AuF_6_)_2_ were partially decomposed at the surface and covered with Ca(AuF_6_)_2_; (c) the Raman spectra really belong to [Ca(HF)_2_](AuF_6_)_2_. We believe that the third option is correct. This is in agreement with the results obtained in other MF_2_/XF_5_ (M = Ca, Sr, Ba; X = As, Sb, Ta, Ru) systems, where the crystallization products from the HF saturated solution always contained HF coordinated to Ca, Sr, or Ba atoms. Some examples are [Ca(HF)_*n*_](AsF_6_)_2_ (*n* = 1, 6),^47^ [Ca(HF)_2_](SbF_6_)_2_,^48^ [Sr(HF)_3_](TaF_6_)_2_,^49^ [Ba(HF)](RuF_6_)_2_,^48^ and [Ba(HF)](AsF_6_)_2_.^50^ To date, no crystals of M(XF_6_)_2_ (M = Ca, Sr, Ba; X = As, Sb, Ta, Ru) have been prepared so far. In some of the Ca^2+^/[AuF_6_]^−^ crystallizations, crystals containing [AuF_4_]^−^ anions were observed (Table S2). The resulting compound has the formula Ca(AuF_4_)(AuF_6_). Some Au^5+^ was reduced upon contact with moisture that can penetrate through the FEP reaction vessel walls. An attempt to grow single crystals of the Ca-salt with a lower content of [AuF_6_]^−^ [i.e., CaFAuF_6_ or Ca[H_*n*_F_*n*+1_](AuF_6_)] failed. At a lower content of [AuF_6_]^−^ (i.e., initial molar ratio Ca^2+^/[AuF_6_]^−^ = 1:1), only [Ca(HF)_2_](AuF_6_)_2_ and [Ca(HF)](AuF_4_)(AuF_6_) were detected in the crystallization products.

In the case of strontium, crystals of [Sr(HF)_2_](AuF_6_)_2_ and [Sr(HF)]_2_(AuF_4_)(AuF_6_)_3_ were grown from solutions in which the Sr^2+^/[AuF_6_]^−^ molar ratio was equal to 1:2 (Table S2). At a molar ratio of 1:1, Sr(H_2_F_3_)(AuF_6_) and [Sr(HF)](H_3_F_4_)(AuF_6_) were observed. In some experiments, single crystals of [O_2_]_2_[Sr(HF)]_5_[AuF_6_]_12_·HF were grown. This was due to the contamination of the reaction mixture with oxygen during the photochemical preparation of the crystallization solution by UV-irradiated F_2_ in aHF.

Crystallizations from Ba^2+^/[AuF_6_]^−^ hydrogen fluoride solutions (molar ratios 1:1 and 1:2) resulted in crystal growth of Ba[Ba(HF)]_6_(AuF_6_)_14_ and [Ba(HF)]_4_(AuF_4_)(AuF_6_)_7_ (Figure S2). When a 1:1 ratio was used, single crystals of Ba(H_3_F_4_)_2_^51^ were also observed.

In an attempt to prepare M(BF_4_)(AuF_6_) salts with mixed anions, a small amount of BF_3_ was added to the crystallization mixture. Single crystals of M(BF_4_)(AuF_6_) (M = Sr, Ba) were successfully grown (Figures S3 and S4), while in the case of calcium, single crystals of [Ca(HF)](BF_4_)_2_ (Figure S5) and a yellow crystalline product of unknown composition (Figure S6) were detected.

### Crystal Structures

The corresponding crystal data and refinement results for [Ca(HF)_2_](AuF_6_)_2_, Ba[Ba(HF)]_6_(AuF_6_)_14_, [Sr(HF)](H_3_F_4_)(AuF_6_), [Ba(HF)]_4_(AuF_4_)(AuF_6_)_7_, M(BF_4_)(AuF_6_) (M = Sr, Ba), [Ca(HF)](BF_4_)_2_, and [O_2_]_2_[Sr(HF)]_5_[AuF_6_]_12_·HF are summarized in Table 1.

**Table 1:** Summary of Crystal Data and Refinement Results of [Ca(HF)_2_](AuF_6_)_2_, Ba[Ba(HF)]_6_(AuF_6_)_14_, [Sr(HF)](H_3_F_4_)(AuF_6_), [Ba(HF)]_4_(AuF_4_)(AuF_6_)_7_, M(BF_4_)(AuF_6_) (M = Sr, Ba), [Ca(HF)](BF_4_)_2_, and [O_2_]_2_[Sr(HF)]_5_[AuF_6_]_12_·HF Compounds

chem. formula	[Ca(HF)_2_]–(AuF_6_)_2_	Ba[Ba(HF)]_6_–(AuF_6_)_14_	[Sr(HF)]–(H_3_F_4_)(AuF_6_)	[Ba(HF)]_4_–(AuF_4_)(AuF_6_)	[Ca(HF)]–(BF_4_)_2_
crystal system	triclinic	rhombohedral	monoclinic	tetragonal	triclinic
space group	*P*1̅	*R*3̅	*P*2_1_/*m*	*I*4_1_/*a*	*P*1̅
*a* (Å)	5.6598(3)	19.8309(6)	5.8708(2)	11.1380(3)	5.1827(4)
*b* (Å)	8.8838(7)	19.8309(6)	8.1261(3)	11.1380(3)	6.5414(6)
*c* (Å)	10.1017(5)	14.2361(4)	8.6531(3)	32.0799(11)	9.7870(7)
α (deg)	93.884(5)	90	90	90	108.679(7)
β (deg)	91.773(4)	90	92.399(4)	90	91.057(6)
γ (deg)	97.742(5)	120	90	90	93.386(7)
*V* (Å^3^)	501.72(5)	4848.5(3)	412.45(3)	3979.7(3)	313.53(5)
*Z*	2	3	2	4	2
*T* (K)	150	150	150	150	150
*R*_1_^a^	0.0398	0.0410	0.0225	0.0263	0.0535
w*R*_2_^b^	0.1051	0.1204	0.0539	0.0601	0.1404

chem. formula	α-Sr(BF_4_)–(AuF_6_)	β-Sr(BF_4_)–(AuF_6_)	Ba(BF_4_)–(AuF_6_)^c^	[O_2_]_2_[Sr(HF)]_5_–[AuF_6_]_12_·HF
crystal system	monoclinic	orthorhombic	orthorhombic	monoclinic
space group	*P*2_1_/*c*	*Pnma*	*Pnma*	*P*2_1_/*n*
*a* (Å)	6.1193(3)	10.2969(5)	10.4846(3)	21.8471(10)
*b* (Å)	11.4512(6)	6.1506(4)	6.4416(2)	10.5392(4)
*c* (Å)	10.2122(5)	11.4529(6)	11.7209(4)	27.0291(12)
α (deg)	90	90	90	90
β (deg)	90.106(4)	90	90	104.565(5)
γ (deg)	90	90	90	90
*V* (Å^3^)	715.60(6)	725.34(7)	791.60(4)	6023.5(5)
*Z*	1	4	4	4
*T* (K)	150	293	150	150
*R*_1_^a^	0.0245	0.0298	0.0275	0.0409
w*R*_2_^b^	0.0470	0.0731	0.0646	0.0911

a *R*_1_ = Σ∥*F*_o_| – |*F*_c_∥/Σ|*F*_o_| for *I* > 2σ(*I*).

b w*R*_2_ = [Σ[*w*(*F*_o_^2^ – *F*_c_^2^)^2^]/Σ*w*(*F*_o_^2^)^2^]^1/2^.

c The crystal structures determined at 80 and 296 K are the same as at 150 K.

### Crystal Structure of [Ca(HF)_2_](AuF_6_)_2_

**Figure 2:**
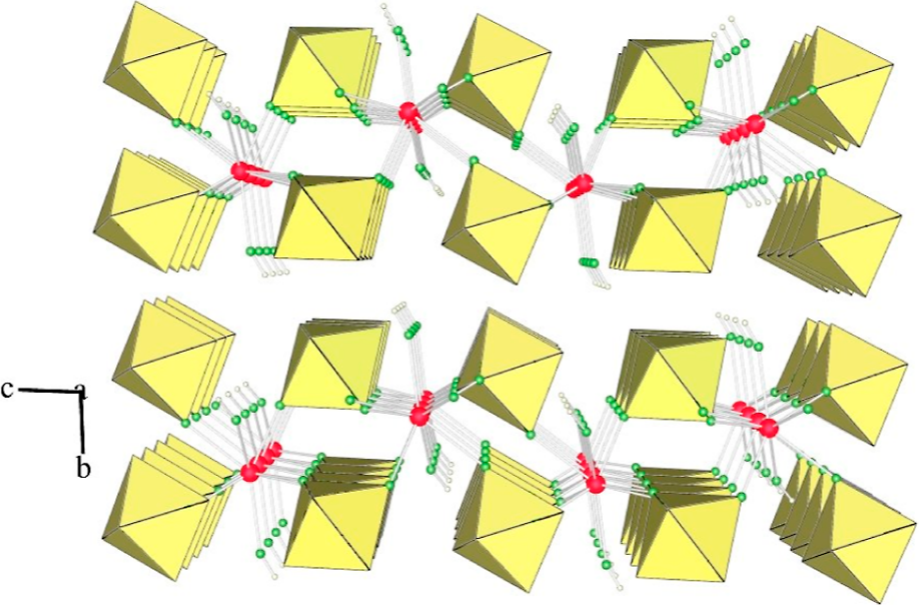
Packing of slabs in the crystal structure of [Ca(HF)_2_](AuF_6_)_2_ (yellow octahedra: AuF_6_; red circles: Ca; green circles: F; small colorless circles: H).

[Ca(HF)_2_](AuF_6_)_2_ crystallizes in the triclinic space group (*P*1̅, *Z* = 2 at 150 K) and is not isotypic with [Ca(HF)_2_](SbF_6_)_2_ (monoclinic, *P*2_1_/*n* at 200 K).^47^ [Ca(HF)_2_](AuF_6_)_2_ has a layered structure in which Ca atoms are linked by AuF_6_ units (Figure 2).

**Figure 3:**
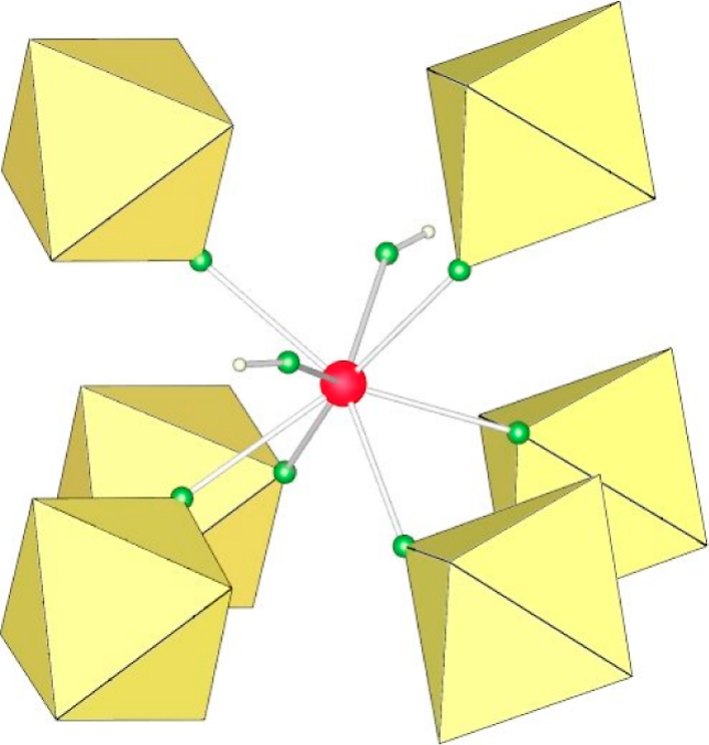
Coordination of Ca^2+^ atoms in the crystal structure of [Ca(HF)_2_](AuF_6_)_2_.

Eight fluorine atoms provided by six AuF_6_ units and two HF molecules coordinate each of the crystallographically distinct Ca atoms (Figure 3). The Ca–F(−H) distances are 2.371(6) and 2.415(7) Å, and the Ca–F(−AuF_5_) bond lengths range from 2.302(6) to 2.443(6) Å. The corresponding bond lengths in [Ca(HF)_2_](SbF_6_)_2_, where Ca is also eight-coordinated, are 2.304(3)/2.315(3) Å for Ca–F(−H) and 2.328(3)–2.430(3) Å for Ca–F(−SbF_5_).^47^ There are two crystallographically nonequivalent Au atoms (Figure S7). The Au–F_b_ bond lengths between gold and the bridging fluorine atoms (Au–F_b_–Ca) are elongated (1.913(6)–1.929(5) Å) compared to the Au–F_t_ bonds between gold and the terminal fluorine atoms (F_t_: 1.871(6)–1.887(6) Å). Two of the latter are slightly elongated (1.883(5) and 1.887(6) Å), which is due to hydrogen bonding with the HF molecule. There are intralayer and interlayer F–H···F–AuF_5_ hydrogen bonds (the F···F distances for the former are 2.676(9) and 2.713(8) Å for the latter).

### Crystal Structure of Ba[Ba(HF)]_6_(AuF_6_)_14_

**Figure 4:**
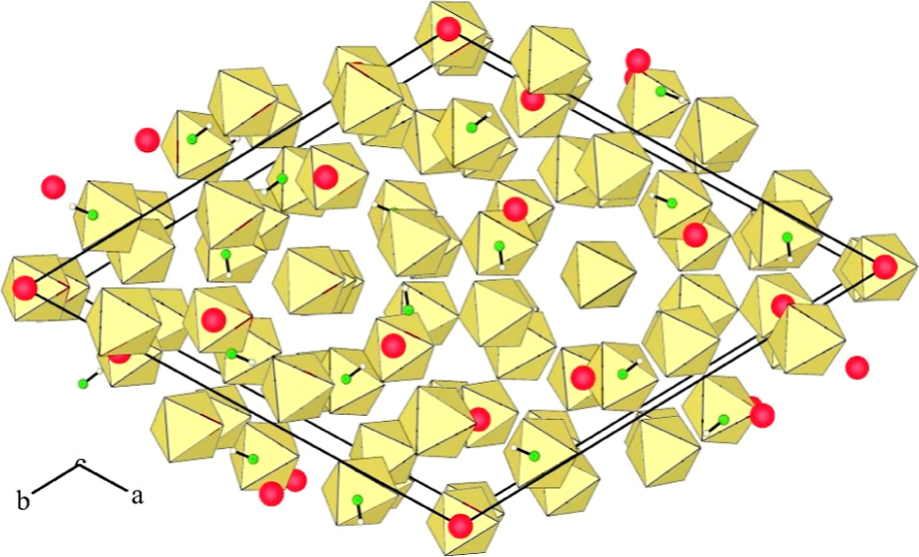
Rhombohedral unit cell and packing of cations, anions, and HF molecules in the crystal structure of Ba[Ba(HF)]_6_(AuF_6_)_14_. For clarity, Ba–F bonds are not shown (yellow octahedra: AuF_6_; red circles: Ba; green circles: F; small colorless circles: H).

**Figure 5:**
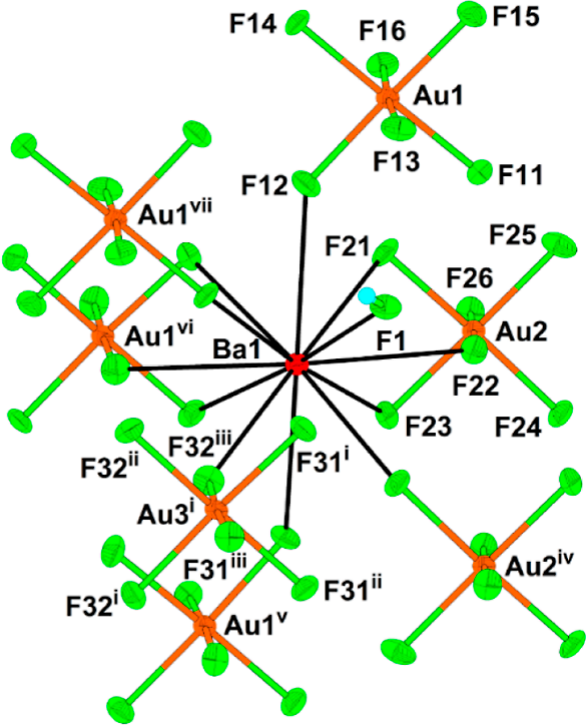
12-fold coordination of the Ba1 atom in the crystal structure of Ba[Ba(HF)]_6_(AuF_6_)_14_. The thermal ellipsoids are drawn at the 50% probability level. The symmetry operations are (i) 1/3 + *x*, −1/3 + *y*, −1/3 + *z*; (ii) 4/3 – *y*, 2/3 + *x* – y, −1/3 + *z*; (iii) 1/3 – *x* + *y*, 2/3 – *x*, −1/3 + *z*; (iv) 1 – *x*, 1 – *y*, 1 – *z*; (v) −1/3 + *y*, 1/3 – *x* + *y*, 4/3 – *z*; (vi) 2/3 – *x* + *y*, 4/3 – *x*, 1/3 + *z*; (vii) 5/3 – *x*, 4/3 – *y*, 4/3 – *z*.

**Figure 6:**
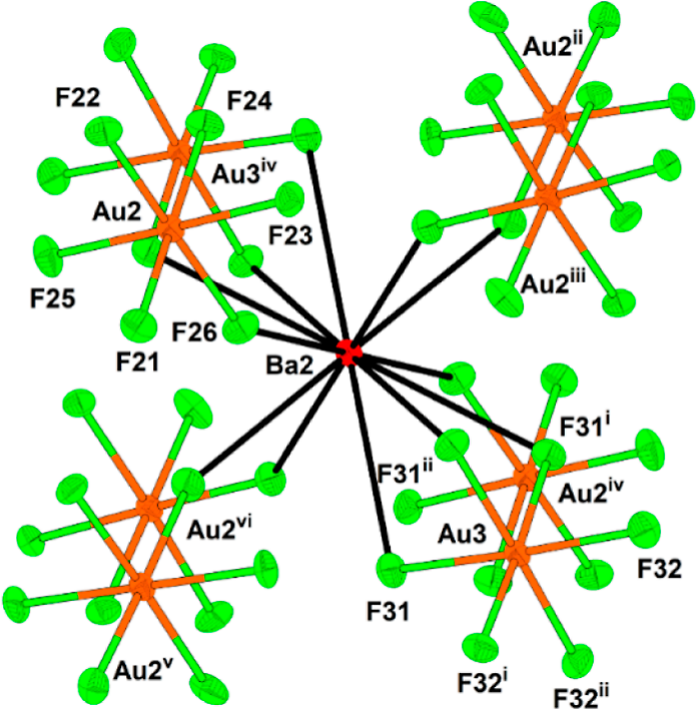
12-fold coordination of the Ba2 atom in the crystal structure of Ba[Ba(HF)]_6_(AuF_6_)_14_. The thermal ellipsoids are drawn at the 50% probability level. The symmetry operations are (i) 1 – *x*, 1 + *x* – *y*, *z*; (ii) −*x* + *y*, 1 – *x*, *z*; (iii) −1/3 + *y*, 1/3 – *x* + *y*, 4/3 – *z*; (iv) 2/3 – *x*, 4/3 – *y*, 4/3 – *z*; (v) 2/3 + *x* – y, 1/3 + *x*, 4/3 – *z*; (vi) 1 – *y*, 1 + *x* – *y*, *z*.

The crystal structure of Ba[Ba(HF)]_6_(AuF_6_)_14_ shows a complex three-dimensional network (Figure 4) consisting of two crystallographically distinct Ba atoms (Figures 5 and 6, and S8), three crystallographically independent AuF_6_ groups (Figure S9), and HF molecules bonded to Ba atoms via their F atom.

The peculiarity is that one of the crystallographically unique Ba atoms (Ba1) is coordinated by a HF molecule, and the other (Ba2) is not. The Ba1/Ba2 ratio is 1:6, and the chemical formula is therefore Ba[Ba(HF)]_6_(AuF_6_)_14_. Classical chemical elemental analysis of such a salt would give the formula Ba(AuF_6_)_2_·0.86HF and based on this formula one would expect some kind of HF-solvate to form. In such cases, only determination of the crystal structure can reveal the true nature of such a compound. Both Ba atoms are coordinated 12-fold by fluorine atoms provided by seven AuF_6_ units and one HF molecule for Ba1 (Figure 5) and eight AuF_6_ units for Ba2 (Figure 6). The Ba–F(AuF_5_) bonds range from 2.748(7) to 3.009(8) Å, while the Ba–F(H) bond is the shortest (2.703(7) Å) and thus the strongest of all Ba–F bonds. The F–H···F–AuF_5_ hydrogen bond is quite strong with H···F and F···F distances of 1.74(4) and 2.614(10) Å, respectively.

### Crystal Structure of [Sr(HF)](H_3_F_4_)(AuF_6_)

**Figure 7:**
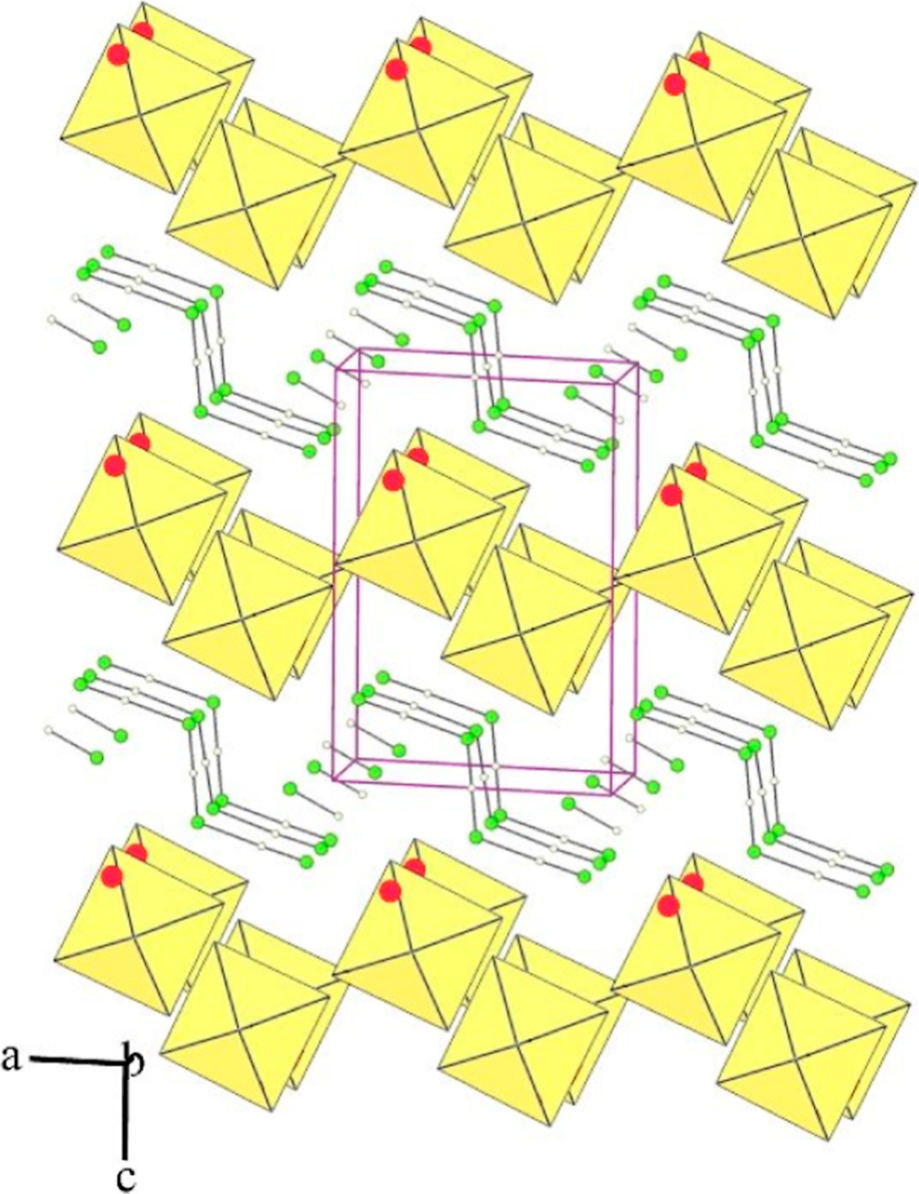
Monoclinic unit cell and packing of Sr^2+^ cations, [AuF_6_]^−^ and [H_3_F_4_]^−^ anions, and HF molecules in the crystal structure of [Sr(HF)](H_3_F_4_)(AuF_6_). For clarity, the Sr–F bonds are not shown (yellow octahedra: AuF_6_; red circles: Sr; green circles: F; small colorless circles: H).

The crystal structure of [Sr(HF)](H_3_F_4_)(AuF_6_) crystallizes in the monoclinic space group *P*2_1_/*m*. The crystal structure consists of a three-dimensional framework of Sr^2+^ cations associated with [AuF_6_]^−^ and [H_3_F_4_]^−^ anions and HF molecules (Figure 7).

**Figure 8:**
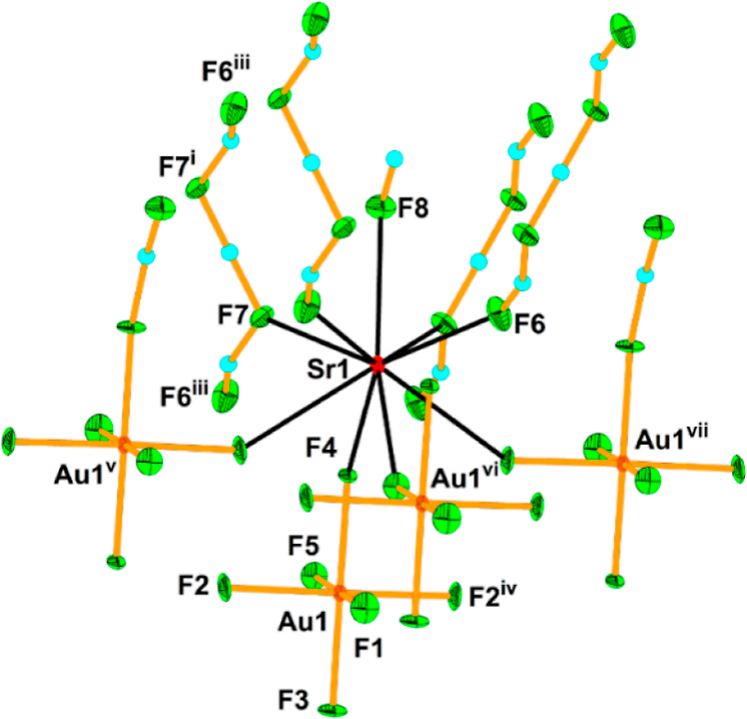
Ninefold coordination of the Sr atom in the crystal structure of [Sr(HF)](H_3_F_4_)(AuF_6_). The thermal ellipsoids are drawn at the 50% probability level. The symmetry operations are (i) 1 – *x*, 1 – *y*, 2 – *z*; (ii) −*x*, 1/2 + *y*, 2 – *z*; (iii) 1 + *x*, 1/2 – *y*, *z*; (iv) *x*, 1/2 – *y*, *z*; (v) 1 – *x*, 1/2 + *y*, 1 – *z*; (vi) −1 + *x*, *y*, *z*; (vii) 1 – *x*, −1/2 + *y*, 1 – *z*.

The coordination of the Sr atom consists of nine fluorine atoms provided by four [AuF_6_]^−^, four [H_3_F_4_]^−^ anions, and one HF molecule (Figures 8 and S10). The Sr–F(AuF_5_) bonding contacts are in the range of 2.565(4)–2.612(3) Å. The bond contacts between the Sr atom and the F atoms of the [H_3_F_4_]^−^ anions are shorter (2.490(3)–2.505(3) Å), while the Sr–F(H) bond distance (2.522(5) Å) lies between these values. The reported Sr–F(H) bond lengths in the crystal structure of Sr(HF)_3_(TaF_6_)_2_ (200 K) have similar values (2.51(2)–2.55 (2) Å).^48^

In the octahedral AuF_6_ unit, one Au–F(H) and four Au–F_b_(−Sr) (Figure S11) are in the range of 1.880(3)–1.905(4) Å, while the sixth Au–F_t_ bond is shorter (1.866(4) Å). The HF molecule bonded to the Sr atom is involved in hydrogen bonding with the F atom of the AuF_6_ unit (Figure S11). The F···F distance between the F atoms involved in the hydrogen bond is 2.596(6) Å.

**Figure 9:**
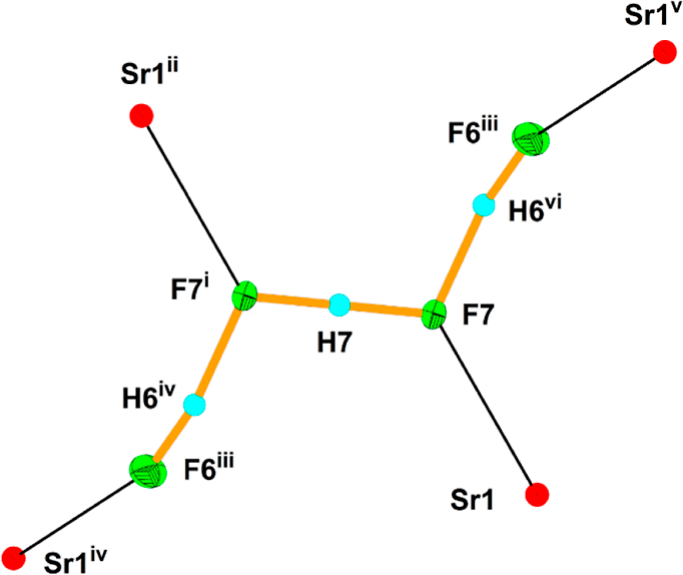
[H_3_F_4_]^−^ anion and its interactions with Sr^2+^ cations in the crystal structure of [Sr(HF)](H_3_F_4_)(AuF_6_). The thermal ellipsoids are drawn at the 50% probability level. The symmetry operations are (i) 1 – *x*, 1 – *y*, 2 – *z*; (ii) 1 – *x*, 1/2 + *y*, −*z*; (iii) 1 + *x*, 1/2 – *y*, *z*; (iv) −*x*, 1/2 + *y*, 2 – *z*; (v) 1 + *x*, *y*, *z*; (vi) 1 + *x*, 1/2 – *y*, *z*.

In the [H_3_F_4_]^−^ anion (Figure 9), the H6 atoms are located closer to the two terminal F atoms (F6; 1.02 Å) than to the central ones (F7; 1.45 Å). The H7 atom is located in the middle of the F7 atoms (1.150 Å). Therefore, the appropriate structural formula is [(FH)(FHF)(HF)]^−^. Previously reported [H_3_F_4_]^−^ isomers have branched- or linear-chain geometry.^50,52−55^ The linear [H_3_F_4_]^−^ isomers in KF·2.5HF^51^ and [Sr(HF)](H_3_F_4_)(AuF_6_) have different conformations. The former has a U-shaped conformation,^51^ while the latter has a Z-shaped conformation (Figure 9).

### Crystal Structure of [Ba(HF)]_4_(AuF_4_)(AuF_6_)_7_

**Figure 10:**
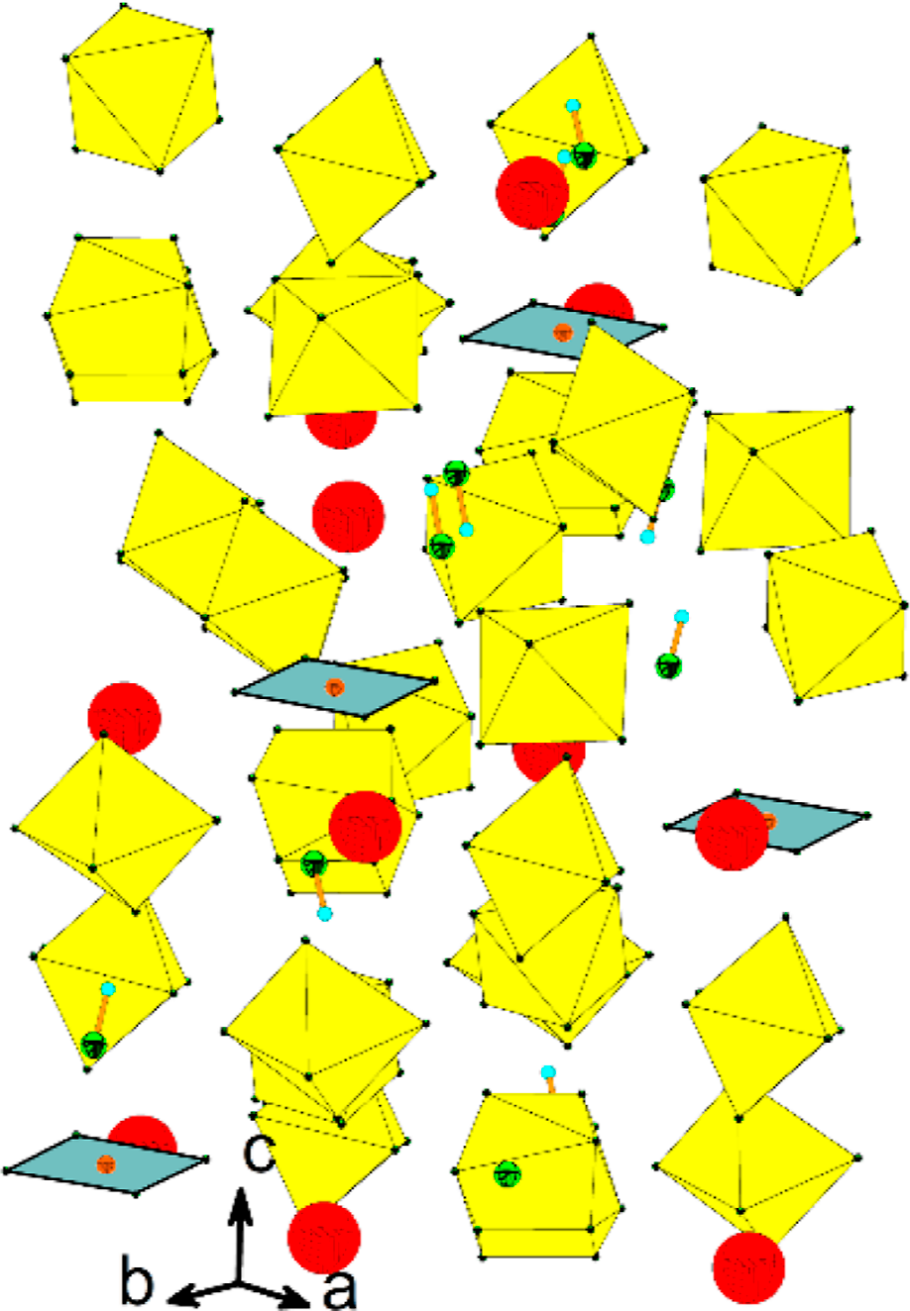
Packing of Ba^2+^ cations, [AuF_6_]^−^ and [AuF_4_]^−^ anions, and HF molecules in the crystal structure of [Ba(HF)]_4_(AuF_4_)(AuF_6_)_7_. For clarity, the Ba–F bonds are not shown. Only one position is shown for each disordered F atom (yellow octahedra: AuF_6_; blue plaques: AuF_4_; red circles: Ba; green circles: F; small blue circles: H).

The crystal structure of [Mg(HF)](AuF_4_)(AuF_6_)^36^ was the first example of a [AuF_4_]^−^/[AuF_6_]^−^ salt with mixed anions, while the crystal structure of [Ba(HF)]_4_(AuF_4_)(AuF_6_)_7_ is the second example. It consists of crystallographically unique Ba^2+^ cations connected by three crystallographically independent AuF_6_ octahedra, as well as unique AuF_4_ plaques and HF molecules (Figures 10 and S12).

**Figure 11:**
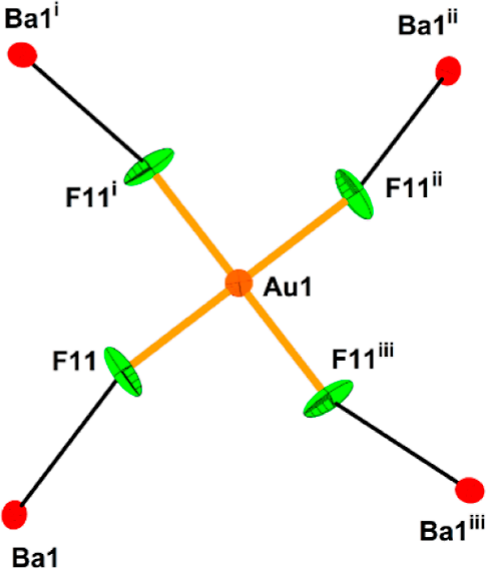
[AuF_4_]^−^ anion and its interactions with Ba^2+^ cations in the crystal structure of [Ba(HF)]_4_(AuF_4_)(AuF_6_)_7_. The thermal ellipsoids are drawn at the 50% probability level. The symmetry operations are (i) 3/4 + *y*, 5/4 – *x*, 5/4 – *z*; (ii) 2 – *x*, 1/2 – *y*, *z*; (iii) 5/4 – *y*, −3/4 + *x*, 5/4 – *z*. Crystal structures of M(BF_4_)(AuF_6_) (M = Sr, Ba).

The fluorine atoms of two AuF_6_ units (Au3 and Au4) are strongly disordered at several positions. In the case of the Au(3)F_6_ unit, two cis-located fluorine centers bonded to Ba cations are ordered, while each of the other four F atoms is distributed in three positions (looks like a whole octahedron—with the exception of bridging fluorines—wobble). An even greater disorder was observed for the Au(4)F_6_ unit: six fluorine atoms are distributed over 24 positions. For this reason, the detailed discussion of some bond distances is not very realistic. Six AuF_6_ units give eight fluorine ligands bonded to one Ba atom. Two F atoms, one of the AuF_4_ units, and one of the HF molecules complete the 10-fold coordination of Ba^2+^ (Figure S13). Each of the fluorine atoms of the [AuF_4_]^−^ anion serves as a bridging ligand connecting the gold atom (Au1) to the Ba atom (Figure 11). The Au–F bond lengths in the AuF_4_ unit (4 × 1.900(5) Å) are comparable to those observed in [Mg(HF)](AuF_4_)(AuF_6_) (1.898(7)–1.916(7) Å).^36^ The HF molecule is coordinated to the Ba atom through its fluorine atom. The hydrogen atom is involved in a hydrogen bond with the F atom of the AuF_6_ group (Au3).

### Crystal Structures of M(BF_4_)(AuF_6_) (M = Sr, Ba).

**Figure 12:**
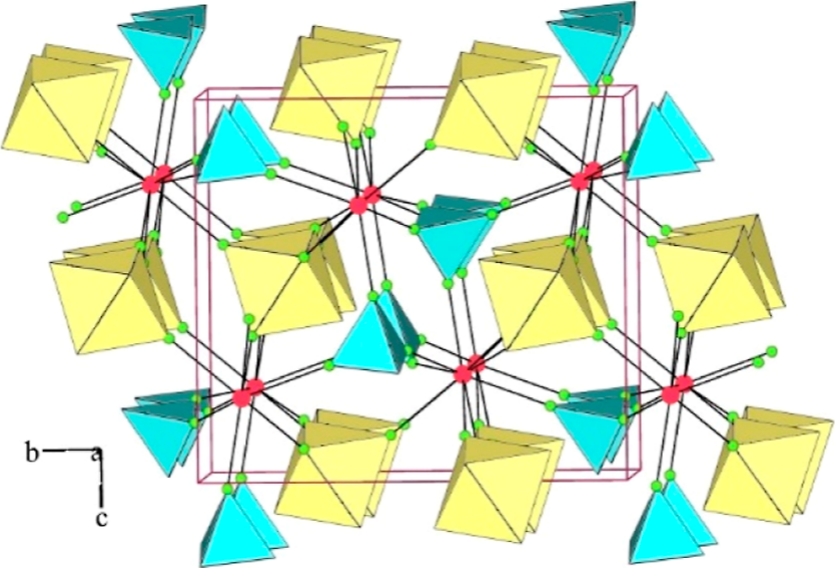
Packing of Sr^2+^ cations and [AuF_6_]^−^ and [BF_4_]^−^ anions in the crystal structure of monoclinic α-Sr(BF_4_)(AuF_6_) (yellow octahedra: AuF_6_; blue tetrahedra: BF_4_; red circles: Sr; green circles: F).

Sr(BF_4_)(AuF_6_) crystallizes in two modifications (Figures 12 and S14). A high-temperature β-phase (structure determined at 293 K) crystallized in an orthorhombic unit cell, and a low-temperature α-phase (structure determined at 150 K) crystallized in a monoclinic unit cell (Table 1). No phase transition was observed for Ba(BF_4_)(AuF_6_) in the range of 80–230 K. Only one orthorhombic modification was observed, which is isotypic to the high-temperature modification of the Sr salt. This type is isotypic to the already known Ba(BF_4_)(AsF_6_) (150 and 200 K).^53^ Both the monoclinic and orthorhombic modifications have similar features; that is, their crystal structure is a 3-D framework consisting of the M^2+^ cations (M = Sr, Ba) connected by AuF_6_ octahedra and BF_4_ tetrahedra (Figure 12). Since this type of structure has already been well described,^53^ it will not be discussed in too much detail here.

In both modifications, the M^2+^ cations are ninefold coordinated by fluorine atoms provided by five AuF_6_ groups and four BF_4_ groups (Figures S15 and S16). In the β-phase of Sr(BF_4_)(AuF_6_), the AuF_6_^–^ anion exhibits a twofold rotational disorder of the terminal fluorine positions about the F(4)–Sb–F(5) axis with equal occupancy for both orientations. The same type of AuF_6_^–^ disorder was observed in the crystal structure of the Ba(BF_4_)(AuF_6_) salt. In the latter, three atoms of the BF_4_ unit are disordered over two positions.

### Crystal Structure of [Ca(HF)](BF_4_)_2_

**Figure 13:**
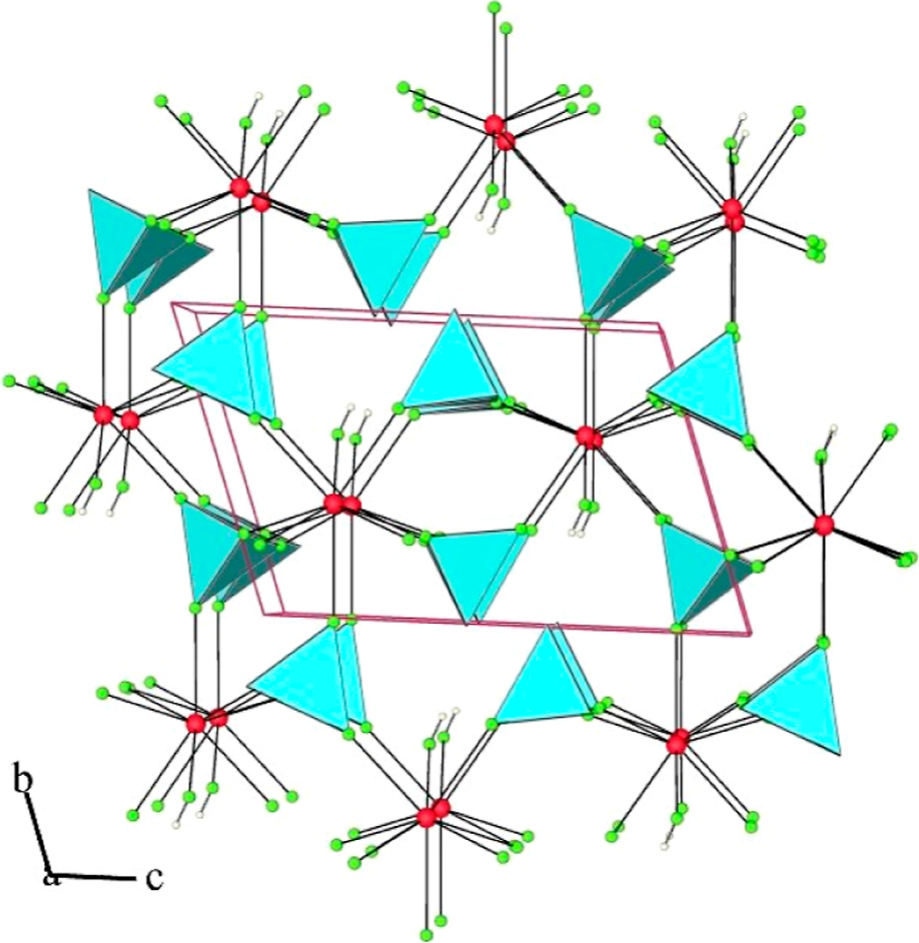
Packing of Ca^2+^ cations, [BF_4_]^−^ anions, and HF molecules in the crystal structure of [Ca(HF)](BF_4_)_2_ (blue tetrahedra: BF_4_; red circles: Sr; green circles: F; small colorless circles: H).

Single crystals of [Ca(HF)](BF_4_)_2_ were obtained in an attempt to grow crystals of Ca(BF_4_)(AuF_6_). In [Ca(HF)](BF_4_)_2_, the molecule HF is coordinated to the Ca atom through its fluorine atom (Figure 13). An earlier example of this type was [Sr(HF)](BF_4_)_2_.^48^ It is not isotypic to the Ca-salt.

**Figure 14:**
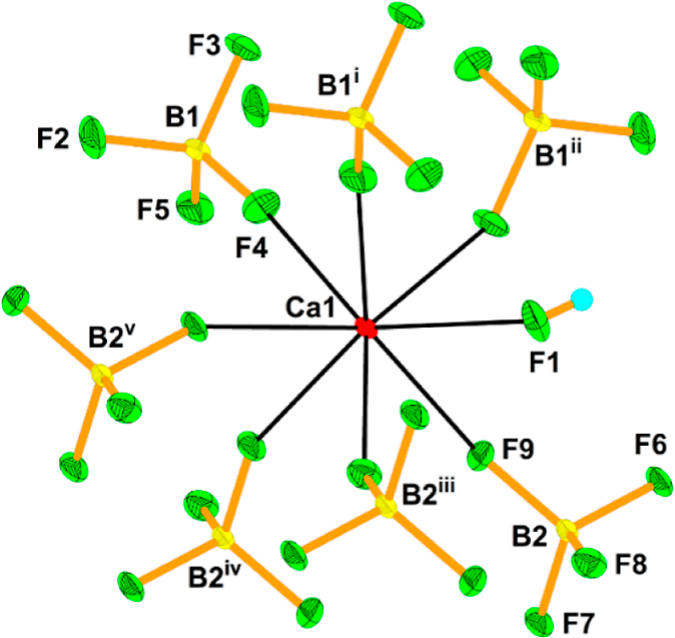
Eightfold coordination of the Ca atom in the crystal structure of [Ca(HF)](BF_4_)_2_. The thermal ellipsoids are drawn at the 50% probability level. The symmetry operations are (i) 1 + *x*, *y*, *z*; (ii) 1 – *x*, 1 – *y*, 1 – *z*; (iii) 2 – *x*, 1 – *y*, −z; (iv) 1 – *x*, 1 – *y*, −*z*; (v) *x*, −1 + *y*, *z*. Crystal structure of [O_2_]_2_[Sr(HF)]_5_[AuF_6_]_12_·HF.

In [Ca(HF)](BF_4_)_2_, the Ca atom is coordinated by eight fluorine atoms of seven BF_4_ units and one HF ligand (Figures 14 and S17). The coordination number of the Ca metal in pure Ca(BF_4_)_2_ is the same (Figure S17), but, of course, the fluorine atoms are provided only by BF_4_ units.^56^ The Ca–F(H) bond distance is longer (2.428(2) Å) than the Ca–F(−BF_3_) bond lengths (2.322(2)–2.365(2) Å). The B–F_b_ bond lengths between boron and the bridging fluorine atoms are longer (1.384(4)–1.398(4) Å) compared to the B–F_t_ bonds between boron and the terminal fluorine atom (1.379(4) Å) involved in hydrogen bonding with the HF molecule (Figure S18). This hydrogen bonding appears to be quite strong, with F···H and F···F distances of 1.90(8) and 2.507(3) Å, respectively.

### Crystal Structure of [O_2_]_2_[Sr(HF)]_5_[AuF_6_]_12_·HF

**Figure 15:**
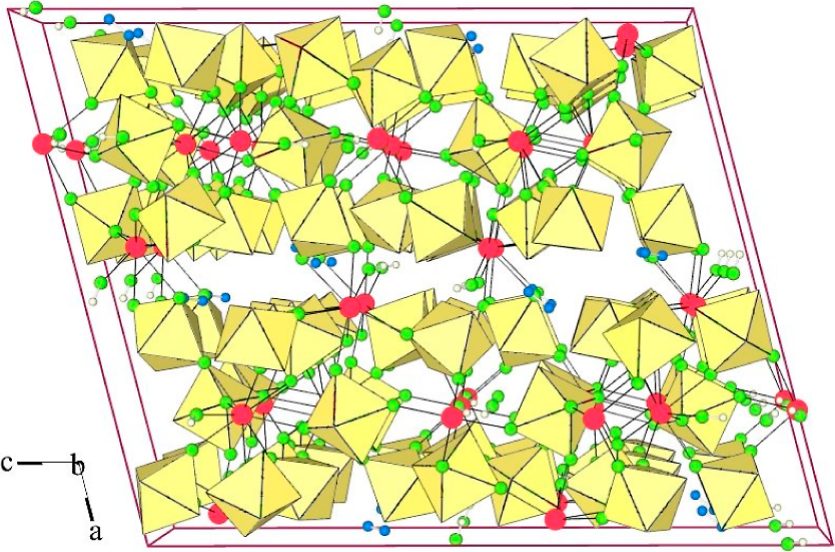
Packing of [Sr(HF)]^2+^ and O_2_^+^ cations, [AuF_6_]^−^ anions, and HF molecules in the crystal structure of [O_2_]_2_[Sr(HF)]_5_[AuF_6_]_12_·HF (yellow octahedra: AuF_6_; red circles: Sr; green circles: F; blue circles: O; small colorless circles: H).

Single crystals of [O_2_]_2_[Sr(HF)]_5_[AuF_6_]_12_·HF were accidentally grown in an attempt to grow single crystals of Sr^2+^/[AuF_6_]^−^ salts (Table S1). Their crystal structure can be described as a 3-D framework consisting of [Sr(HF)]^2+^ cations linked by [AuF_6_]^−^ anions. The O_2_^+^ cations and the non-coordinated HF molecules are located inside the cavities (Figure 15). The packing of Sr^2+^ cations and [AuF_6_]^−^ anions in [O_2_]_2_[Sr(HF)]_5_[AuF_6_]_12_·HF is very similar to the packing of Sr^2+^ cations and [AsF_6_]^−^ anions in [Co(HF)_2_]Sr[Sr(HF)]_2_[Sr(HF)_2_]_2_[AsF_6_]_12_ (abbreviated as CoSr_5_(AsF_6_)_12_·8HF; Figure S19). The unit-cell parameters of the latter are also very close to those of [O_2_]_2_[Sr(HF)]_5_[AuF_6_]_12_·HF.^57^

There are five crystallographically independent Sr^2+^ cations (Figure S20) and 12 crystallographically unique AuF_6_ units. Each of the Sr atoms is ninefold coordinated by fluorine atoms of one HF molecule and six or eight AuF_6_ units. The Sr–F bond lengths (2.428(7)–2.751(8) Å) are in the same range as in [Sr(HF)](H_3_F_4_)(AuF_6_) and the Au–F bond distances (1.853(8)–1.914(7) Å) are comparable to those in other [AuF_6_]^−^ salts. The O–O bond lengths (1.03(1) and 1.01(1) Å) are close to those determined in other O_2_^+^-salts.^58^

## Conclusions

Attempts to grow solvent-free single crystals of M(AuF_6_)_2_ salts (M = Ca, Sr, Ba)^38,39^ from anhydrous hydrogen fluoride (aHF) solutions failed. Instead, crystals of M^2+^ salts with coordinated HF (via the F atom) were obtained. From the comparison of the previously reported Raman spectra of M(AuF_6_)_2_^38,39^ with the spectra recorded on single crystals (Figures S1 and S2), it can be concluded that the former (which were also prepared in aHF), do not correspond to this chemical formula. The report on the M(AuF_6_)_2_ salts appeared in 1990.^38^ At that time, it was already known that binary fluorides, when dissolved in superacids (i.e., aHF acidified with AsF_5_, SbF_5_, etc.), gave solutions of solvated [M(HF)_*n*_]^*x*+^ cations and [XF_6_]^−^ anions (X = As, Sb, etc.),^59,60^ but examples of [M(HF)_*n*_]^*x*+^([XF_6_]^−^)_*x*_ salts in the condensed state were not known. It was assumed that HF is too weak a coordinating ligand for such salts to be stabilized in the solid state. The crystal structure of La(HF)_2_(AsF_6_)_3_ (published in 1998) was the first example in which HF was coordinated directly to a metal center via its F atom.^61^ Later, many more examples followed, and today, it is a well-known fact that crystallizations from aHF solutions can give [M(HF)_*n*_]^*x*+^ salts.^48,56^ In 2000, two different types of Raman spectra (types A and B) were reported for Ba(AuF_6_)_2_, depending on whether KrF_2_ or UV-irradiated F_2_ was used to fluorinate a BaF_2_/2AuF_3_ mixture in aHF.^39^ It was suggested that one of the phases might contain [Ba(HF)_*n*_]^2+^ or [Ba(KrF_2_)_*n*_]^2+^. Since the recent Raman spectra recorded on single crystals of Ba[Ba(HF)]_6_(AuF_6_)_14_ (in the preparation of which KrF_2_ was not used) are in agreement with the reported spectra,^39^ the latter possibility is now ruled out. Here, we must mention that the existence of M^II^(Au^V^F_6_)_2_·*n*KrF_2_ (M^II^ = Ca, Sr, Ba; *n* = 0–4) compounds has been reported in the past.^41,62−65^ They are reported to be stable at room temperature and have been partially characterized. They were described as clathrates, but the true nature of these compounds was never established. The first real evidence for the existence of compounds with a KrF_2_ ligand coordinated to a metal center (e.g., [Mg(KrF_2_)_4_(AsF_6_)_2_] and [Mg(KrF_2_)_4_(AsF_6_)_2_]·2BrF_5_) was reported in 2017.^66^

Another problem in the synthesis of [AuF_6_]^−^ salts is the possible formation of byproducts, such as [O_2_]^+^ salts (i.e., O_2_AuF_6_). Their presence can lead to more complex compounds, for example, [O_2_]_2_[Sr(HF)]_5_[AuF_6_]_12_·HF. The second problem is the extreme sensitivity of Au(V) compounds to moisture or other impurities, which can lead to partial reduction of Au(V) to Au(III) and the formation of mixed-anion salts [AuF_4_]^−^/[AuF_6_]^−^.

Attempts to prepare MFAuF_6_ salts (M = Ca, Sr, Ba) also failed. Instead, [M(HF)_*n*_]^*x*+^ salts with [AuF_6_]^−^ and poly(hydrogen fluoride) anions were obtained [e.g., [Sr(HF)](H_3_F_4_)(AuF_6_)]. When a small amount of BF_3_ is added, [BF_4_]^−^/[AuF_6_]^−^ salts with mixed anions are obtained (M(BF_4_)(AuF_6_), M = Sr, Ba). A similar attempt to synthesize the corresponding Ca-salt gave [Ca(HF)](BF_4_)_2_.

It appears that it is not possible to prepare M(XF_6_)_2_ or MFXF_6_ (M = Ca, Sr, Ba; X = As, Sb, Au, etc.) from aHF solutions. Ca, Sr, and Ba prefer higher coordination numbers. Achieving such a high coordination number with only [XF_6_]^−^ ligands would lead to a very crowded environment, which is energetically less favorable than having some HF in the coordination sphere.

Together with the reported crystal structure, the preliminary results of the crystal structures of Sr(HF)(AuF_6_)_2_, Sr(H_2_F_3_)(AuF_6_), Ca(AuF_4_)(AuF_6_), and [Sr(HF)]_2_(AuF_6_)_3_(AuF_4_) (Figures S21–S24) suggest that more phases can be expected in the M^2+^ (M = Ca, Sr, Ba)/[AuF_6_]^−^ system.

## Experimental Section

CAUTION: aHF and some fluorides are highly toxic and must be handled in a well-ventilated hood. Protective clothing must be worn at all times!

### Materials and Methods

#### Reagents

Commercially available reagents BF_3_ Union Carbide Austria (GmbH, 99.5%), CaF_2_ (Merck), SrF_2_ (Alfa Aesar, 99.99%), and BaF_2_ (Alfa Aesar, 99.995%) were used as supplied. AuF_3_ was synthesized by the reaction of AuCl_3_ (Alfa Aesar, 99.99%) with elemental fluorine F_2_ (Solvay Fluor and Derivate GmbH, 99.98%) in aHF (Linde AG, Pullach, Germany, 99.995%) at room temperature. The “M(AuF_6_)_2_“ salts (M = Ca, Sr, Ba) were prepared as previously described.^39^

#### Synthetic Apparatus

All manipulations were performed under anhydrous conditions. Nonvolatile materials were handled in an M. Braun glovebox in an argon atmosphere in which the amount of water did not exceed 0.5 ppm. Gaseous F_2_ and volatile compounds such as aHF and BF_3_ were handled on a vacuum line constructed from nickel and polytetrafluoroethylene (PTFE).

The vessels used for single-crystal growth were made of tetrafluoroethylene–hexafluoropropylene block-copolymer (FEP; Polytetra GmbH, Germany) tubes. The crystallization vessel consisted of two FEP tubes: one with an inner diameter of 16 mm and an outer diameter of 19 mm and the other with an inner diameter of 4 mm and an outer diameter of 6 mm. Each tube was heat-sealed at one end and connected via linear PTFE connections to a PTFE T-part at 90°. The PTFE valve was attached to the T-part at a 180° angle to the 19 mm o.d. tube. All PTFE parts of the valve were enclosed in brass with threads that prevented deformation of the PTFE parts of the valve and facilitated connection to the reaction vessels and vacuum system. PTFE-coated magnetic stir bars were placed inside the reaction vessels. The temperature gradient between the two arms of the crystallization vessels was maintained by cooling a wider arm of a vessel in the Huber Ministat 230 (to −33 °C).

Before use, all reaction and crystallization vessels were dried under dynamic vacuum and passivated with elemental fluorine F_2_ at 1 bar for 2 h. aHF was treated with K_2_NiF_6_ (Advance Research Chemicals Inc, 99.9%) for several hours before use and stored in FEP vessels over K_2_NiF_6_.

#### Synthesis and Crystal Growth

Solid starting reagents “M(AuF_6_)_2_”^39^ or MF_2_/_*n*_AuF_3_ (M = Ca, Sr, Ba) mixtures were loaded into reaction vessels in a dry box (Table S1 in the Supporting Information). The solvent (HF, 2–6 mL) was condensed to “M(AuF_6_)_2_” (M = Ca, Sr, Ba) at 77 K and warmed to ambient temperature. Similarly, HF (4–6 mL) was condensed onto the MF_2_/*n*AuF_3_ (M = Ca, Sr, Ba; *n* = 1, 2) reaction mixtures. Fluorine was slowly added to the reaction vessel at room temperature until a pressure of 4 bar was reached. In some experiments, a small amount of BF_3_ was added to prepare the mixed-anion salts [BF_4_]^−^/[AuF_6_]^−^. A medium-pressure mercury lamp (Hg arc lamp, 450 W, Ace Glass, USA) was used as the UV source.^39^ The reaction mixture was stirred at room temperature for 1–5 days. After clear yellow solutions were obtained, the fluorine was pumped off. For the purpose of crystallization, the clear solutions were decanted into the 6 mm o.d. sidearm in all cases. Evaporation of the solvent from this side arm was achieved by maintaining a temperature gradient of about 10–20 °C between the two tubes for several weeks. Slow distillation of aHF from the 6 mm o.d. tube into the 19 mm o.d. tube resulted in crystal growth within the 6 mm o.d. tube.

The crystals were treated in different ways. Some crystals were immersed in perfluorodecalin (melting point 263 K) in a dry box, selected under a microscope, and mounted on the goniometer head of the diffractometer in a cold nitrogen stream (265–273 K). Others were sealed in quartz capillaries used for structure determination at room temperature and for recording Raman spectra at several random positions. A special method was used to isolate the crystals of some batches (Table S3). After pumping out the volatiles at low temperature, a small amount (∼0.5–1 mL) of cold (278 K) perfluorinated oil (perfluorodecaline C_10_F_18_) was injected into the narrower FEP tube to cover the crystals. Then, the crystals covered with the cold oil were selected under the microscope and mounted on the goniometer head of the diffractometer in a cold nitrogen stream (265–273 K).

### Characterization Methods

#### Raman Spectroscopy

Raman spectra with a resolution of 0.5 cm^–1^ were recorded at room temperature using a Horiba Jobin Yvon LabRam-HR spectrometer equipped with an Olympus BXFM-ILHS microscope. Samples were excited with the 632.8 nm emission line of a He–Ne laser with a regulated power in the range of 20–0.0020 mW, resulting in 17–0.0017 mW focused through a 50× microscope objective on a 1 μm spot onto the top surface of the sample.

Single crystals or the powdered material were mounted in the glovebox in previously vacuum-dried quartz capillaries, which were first sealed with Halocarbon 25-5S grease (Halocarbon Corp.) inside the glovebox and later heat-sealed in an oxygen–hydrogen flame outside the glovebox.

#### Single-Crystal X-ray Diffraction Analysis

Single-crystal X-ray data for [Ca(HF)_2_](AuF_6_)_2_, Ba[Ba(HF)]_6_(AuF_6_)_14_, Sr(H_3_F_4_)(AuF_6_)·HF, [Ba(HF)]_4_(AuF_4_)(AuF_6_)_7_, M(BF_4_)(AuF_6_) (M = Sr, Ba), [Ca(HF)](BF_4_)_2_, [O_2_]_2_[Sr(HF)]_5_[AuF_6_]_12_·HF, Sr(HF)_2_(AuF_6_)_2_, Sr(H_2_F_3_)(AuF_6_), Ca(AuF_4_)(AuF_6_), and [Sr(HF)]_2_(AuF_6_)_3_(AuF_4_) were collected on a Gemini A diffractometer equipped with an Atlas charge-coupled device (CCD) detector, using graphite monochromated Mo Kα radiation. The data were processed using the CrysAlisPro software suite program package.^67^ Analytical absorption corrections were applied to all data sets. The structure of Sr(H_2_F_3_)(AuF_6_) was solved using the SHELXS program.^68^ All other structures were solved using the dual-space algorithm of the SHELXT^69^ program implemented in the Olex crystallographic software.^70^ Structure refinement was performed using the SHELXL-2014 software.^71^ The hydrogen atoms in the structures of Ba[Ba(HF)]_6_(AuF_6_)_14_ and [Ca(HF)](BF_4_)_2_ were found on difference Fourier maps, and their positional and thermal parameters were freely refined in [Ca(HF)](BF_4_)_2._ In Ba[Ba(HF)]_6_(AuF_6_)_14_, the thermal parameter of the hydrogen atom was constrained to 1.2 U of the connected fluorine atom. The crystals of the salts Sr(HF)(AuF_6_)_2_, Sr(H_2_F_3_)(AuF_6_), Ca(AuF_4_)(AuF_6_), and [Sr(HF)]_2_(AuF_6_)_3_(AuF_4_) were of poor quality or multiple twins. Therefore, only the unit-cell parameters are given (Figures S21–S24). Figures were made with Balls & Sticks^72^ and DIAMOND 4.6 software.^73^

CCDC 2129976 ([Ba(HF)]_4_(AuF_4_)(AuF_6_)_7_), 2129977 ([Ca(HF)_2_](AuF_6_)_2_), 2129978 (α-Sr(BF_4_)(AuF_6_)), 2129979 (β-Sr(BF_4_)(AuF_6_)), 2129980 (Ba[Ba(HF)]_6_(AuF_6_)_14_), 2129981 ([Ca(HF)](BF_4_)_2_), 2129982 ([O_2_]_2_[Sr(HF)]_5_[AuF_6_]_12_·HF), 2129983 (Ba(BF_4_)(AuF_6_)), and 2129984 ([Sr(HF)](H_3_F_4_)(AuF_6_)) contain the supplementary crystallographic data for this work. These data can be obtained free of charge via www.ccdc.cam.ac.uk/data_request/cif, by emailing at data_request@ccdc.cam.ac.uk, or by contacting The Cambridge Crystallographic Data Centre, 12 Union Road, Cambridge CB2 1EZ, UK; fax +44 1223 336033.

## Supporting Information Available

List of known [AuF_6_]^-^ salts, additional experimental details, and some results of characterization by Raman spectroscopy and X-ray diffraction analysis (PDF)
