# Supplementary material for: Crystal Growth from Anhydrous HF Solutions of M2+ (M = Ca, Sr, Ba) and [AuF6]−, Not Only Simple M(AuF6)2 Salts
Source: Inorg Chem. 2022 Jun 30;61(27):10587–97. doi: 10.1021/acs.inorgchem.2c01675 (PMC9377525; doi:10.1021/acs.inorgchem.2c01675)
Supplement: Supplementary file 1 — ic2c01675_si_001.pdf [file ic2c01675_si_001.pdf]

Electronic Supporting Information

Crystal Growth from anhydrous HF solutions of M^{2+} (M = Ca, Sr, Ba) and $[AuF_6]^-$; not only simple $M(AuF_6)_2$ salts

Zoran Mazej,[*,§] Evgeny Goreshnik,[§]

[§]Department of Inorganic Chemistry and Technology, Jožef Stefan Institute, Jamova cesta 39, SI–1000 Ljubljana, Slovenia, zoran.mazej@ijs.si

Table S1. List of known $[AuF_6]^-$ salts with different cations and selected literature.

Compound	Characterization	Ref.
$Xe_2F_{11}AuF_6$ [a]	crystal structure from single crystal data	1
$XeFAuF_6$	Raman spectroscopy	2
Xe_2F_3 AuF_6	Raman spectroscopy	3
XeF_5AuF_6	unit cell from single crystal data, Raman spectroscopy	4
$KrFAuF_6$ [a]	crystal structure from single crystal data, Raman spectroscopy	5
O_2AuF_6 [a]	crystal structure from single crystal data, Raman spectroscopy	5
$NOAuF_6$	IR and Raman spectroscopy	3, 7
ClF_2AuF_6	IR and Raman spectroscopy	6
ClO_2AuF_6	IR and Raman spectroscopy	6
$ClOF_2AuF_6$	IR and Raman spectroscopy	6
ClF_4AuF_6	IR and Raman spectroscopy	6
ClF_6AuF_6	IR and Raman spectroscopy	6
NF_4AuF_6	IR and Raman spectroscopy	7
$N_2F_3AuF_6$	IR and Raman spectroscopy	7
NOF_2AuF_6	IR and Raman spectroscopy	7
BrF_6AuF_6	Mössbauer spectroscopy	8
IF_6AuF_6	X-ray powder diffraction data, Raman spectroscopy	9
$ReF_6^+ReF_7AuF_6^-AuF_5$	Raman spectroscopy	10
$(AgF)_2AgF_4AuF_6$	X-ray powder diffraction data	11
$AgAuF_6$	X-ray powder diffraction data	12
$LiAuF_6$	crystal structure; synchrotron X-ray powder diffraction data	13
$NaAuF_6$	X-ray powder diffraction data, Raman spectroscopy	14, 15
$KAuF_6$ [a]	crystal structure from single crystal data	16
$RbAuF_6$	X-ray powder diffraction data, Raman spectroscopy	14, 15
$CsAuF_6$	X-ray powder diffraction data, Raman spectroscopy	9
$Mg(AuF_6)_2$	X-ray powder diffraction data, Raman spectroscopy	17, 18
$Mg(HF)AuF_4AuF_6$ [a]	crystal structure from single crystal data	16
$Ca(AuF_6)_2$	X-ray powder diffraction data, Raman spectroscopy	17, 18
$Sr(AuF_6)_2$	X-ray powder diffraction data, Raman spectroscopy	17, 18,
$Ba(AuF_6)_2$	X-ray powder diffraction data, Raman spectroscopy	17, 18
$Ni(AuF_6)_2$	X-ray powder diffraction data, Raman spectroscopy	18
$Cu(AuF_6)_2$	X-ray powder diffraction data, Raman spectroscopy	18
$Zn(AuF_6)_2$	X-ray powder diffraction data, Raman spectroscopy	18
$Ag(AuF_6)_2$	X-ray powder diffraction data, Raman spectroscopy	18
$AgFAuF_6$ [a]	crystal structure from single crystal data	19
$Cd(AuF_6)_2$ [a]	crystal structure from single crystal data	16, 18
$Hg(AuF_6)_2$ [a]	crystal structure from single crystal data	19, 18

[a]The crystal structure determined on a single crystal has been reported.

Table S2. Reaction conditions and final products in attempts to grow single crystals of various hexafluoridoaurate(V) salts of alkaline earth metals. The salts of "$M(AuF_6)_2$" (M = Ca, Sr, Ba) were used as starting materials. They were prepared by reactions between MF_2, $2AuF_3$ and KrF_2 or UV-irradiated F_2 in aHF as solvent.[18]

$M(AuF_6)_2$ Experiment label / Year	mass / mg	Synthesis	*V* (aHF) / ml	Solubility in aHF	Time of Cryst. / days	Isolated at:	Observed crystals[a]
Ca-13 2004	~200	UV	5	completely dissolved	30	298 K	$[Ca(HF)_2](AuF_6)_2$
Ca-15 2005	~200	KrF_2	4	partly dissolved	23	298 K	$[Ca(HF)_2](AuF_6)_2$<br>$Ca(AuF_4)(AuF_6)$ [b]
Ca-14 2008	~200	UV	4	partly dissolved	90	298 K	$Ca(AuF_4)(AuF_6)$ [b]
Sr-12 2007	~200	KrF_2	2	partly dissolved	65	298 K	$[Sr(HF)]_2(AuF_6)_3(AuF_4)$ [b]<br>$[Sr(HF)](AuF_6)_2$ [b]
Ba-10 2002	180	UV	2	completely dissolved	11	298 K	$[Ba(HF)]_4(AuF_4)(AuF_6)$
Ba-10 2003	350	KrF_2	6	completely dissolved	28	298 K	$[Ba(HF)]_4(AuF_4)(AuF_6)$
Ba-13 2007	~200	UV	2	completely dissolved	30	298 K	$Ba[Ba(HF)]_6(AuF_6)_{14}$

[a]Numerous crystals from each batch were checked. In some cases, crystals were too small or microcrystalline assemblies were observed that could not be placed on the diffractometer goniometer. [b]Grown crystals were of poor quality.

Table S3. Reaction conditions and final products in the attempts to grow single crystals of various hexafluoridoaurate(V) salts of alkaline earth metals. Reaction mixtures MF_2 (M = Ca, Sr, Ba), $n$$AuF_3$ (n = 1, 2) and F_2 (4 bar) were irradiated with UV light in liquid aHF and allowed to stand until clear yellow solutions were formed. F_2 was then pumped off and crystallizations began.

M	Year	MF_2 / mg	AuF_3	$n[MF_2] : n[AuF_3]$ molar ratio	V (aHF) / ml	Solubility in aHF	Time of Cryst. / days	Isolated at:	Observed crystals[a]
Ca	2017	13	86	1 : 2	4	completely dissolved	43	298 K	$[Ca(HF)_2](AuF_6)_2$
Ca	2019	31	102	1 : 1	6	completely dissolved	51	263 K[b]	$Ca(AuF_4)(AuF_6)$ [c]
Ca	2019	62	202	1 : 1	5	completely dissolved	21	263 K[b]	$[Ca(HF)_2](AuF_6)_2$<br>$Ca(AuF_4)(AuF_6)$[c]
Sr	2017	22	89	1 : 2	4	completely dissolved	23	298 K	$Sr(H_2F_3)(AuF_6)$ [c]<br>$[Sr(HF)](AuF_6)_2$ [c]
Sr	2017	22	89	1 : 2	4	completely dissolved	31	298 K	$[Sr(HF)](AuF_6)_2$ [c]
Sr	2017	22	45	1 : 1	4	completely dissolved	19	273 K[b]	$[Sr(HF)](H_3F_4)(AuF_6)$
Sr	2021	22	89	1 : 2	4	completely dissolved	37	248 K[b]	$(O_2)_2[Sr(HF)_5](AuF_6)_{12}\cdot 6HF$,<br>$O_2AuF_6$, $[Sr(HF)]_2(AuF_6)_3(AuF_4)$[c]
Sr	2021	22	89	1 : 2	4	completely dissolved	74	253 K[b]	$(O_2)_2[Sr(HF)_5](AuF_6)_{12}\cdot 6HF$
Sr	2021	22	45	1 : 1	4	completely dissolved	74	253 K[b]	$(O_2)_2[Sr(HF)_5](AuF_6)_{12}\cdot 6HF$
Ba	2016	23	67	1 : 2	6	completely dissolved	45	298 K	$Ba[Ba(HF)]_6(AuF_6)_{14}$<br>$[Ba(HF)]_4(AuF_4)(AuF_6)_7$
Ba	2017	23	34	1 : 1	4	completely dissolved	12	273 K	$[Ba(HF)]_4(AuF_4)(AuF_6)_7$<br>$Ba(H_3F_4)_2$

[a]A numerber of crystals from each batch were checked. In some cases, the crystals were too small or microcrystalline assemblies were observed that could not be placed on the goniometer of the diffractometer. [b]After the volatiles were pumped out, cooled (278 K) perfluorinated oil was injected into the FEP tube to cover the crystals. [c]Grown crystals were of poor quality.

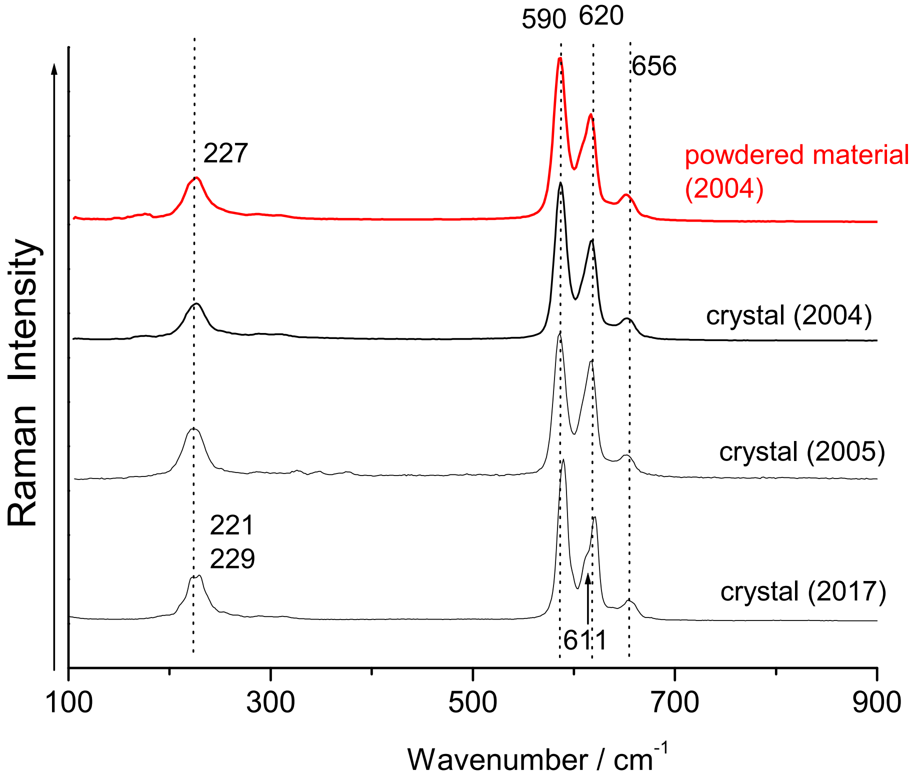

Figure S1. Raman spectrum of powdered $[Ca(HF)_2](AuF_6)_2$[18] and Raman spectra of $[Ca(HF)_2](AuF_6)_2$ (prepared in 2004, 2005 and 2017) recorded on single crystals checked with a diffractometer.

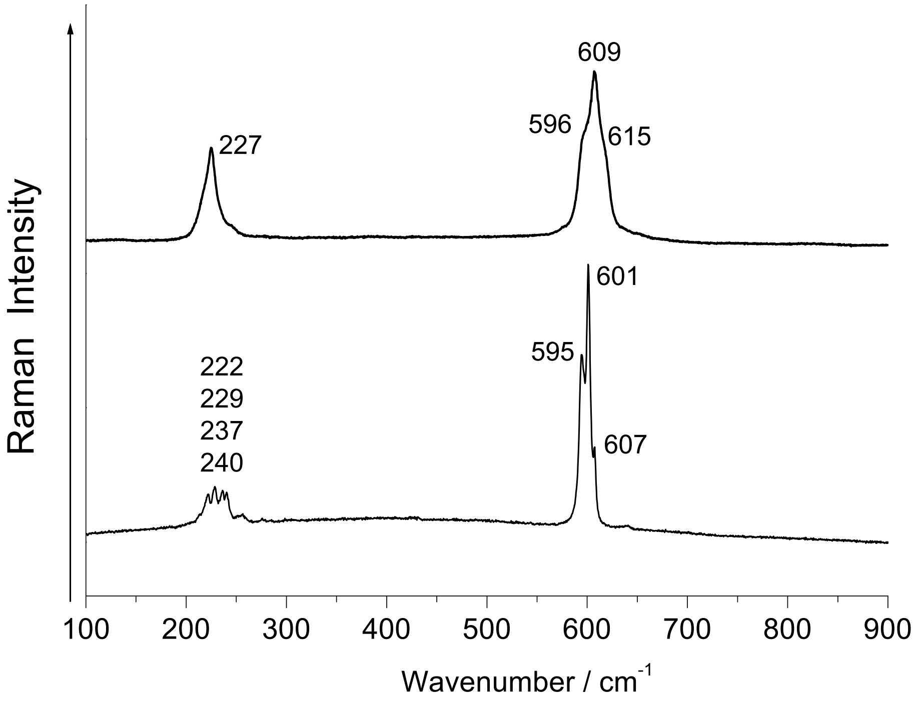

Figure S2. Raman spectra of $[Ba(HF)]_4(AuF_4)(AuF_6)_7$ (top) and $Ba[Ba(HF)]_6(AuF_6)_{14}$ (bottom) recorded on single crystals checked with a diffractometer.

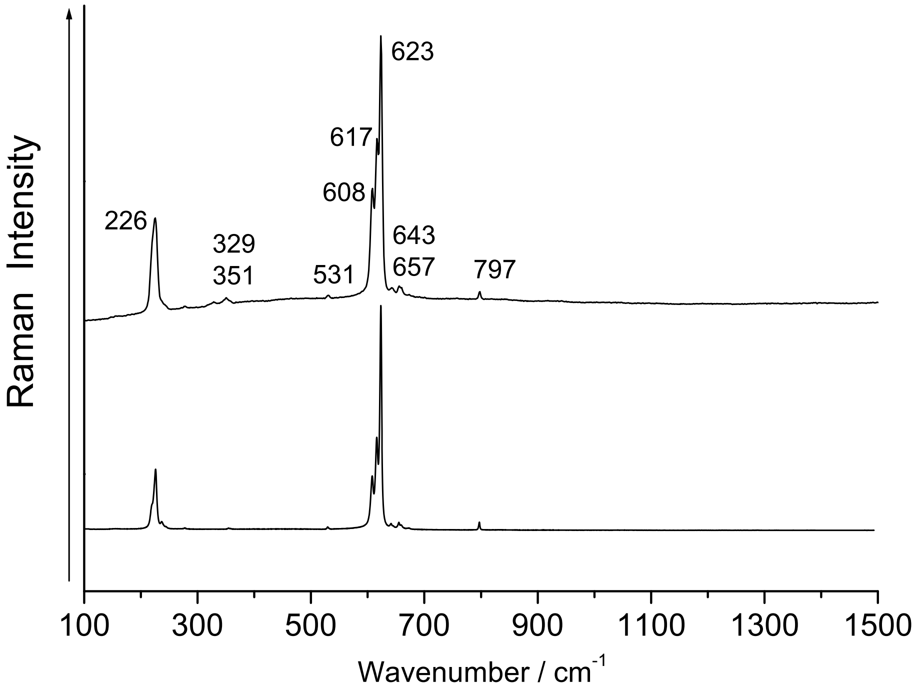

Figure S3. Raman spectra of $Sr(BF_4)(AuF_6)$ recorded on different single crystals.

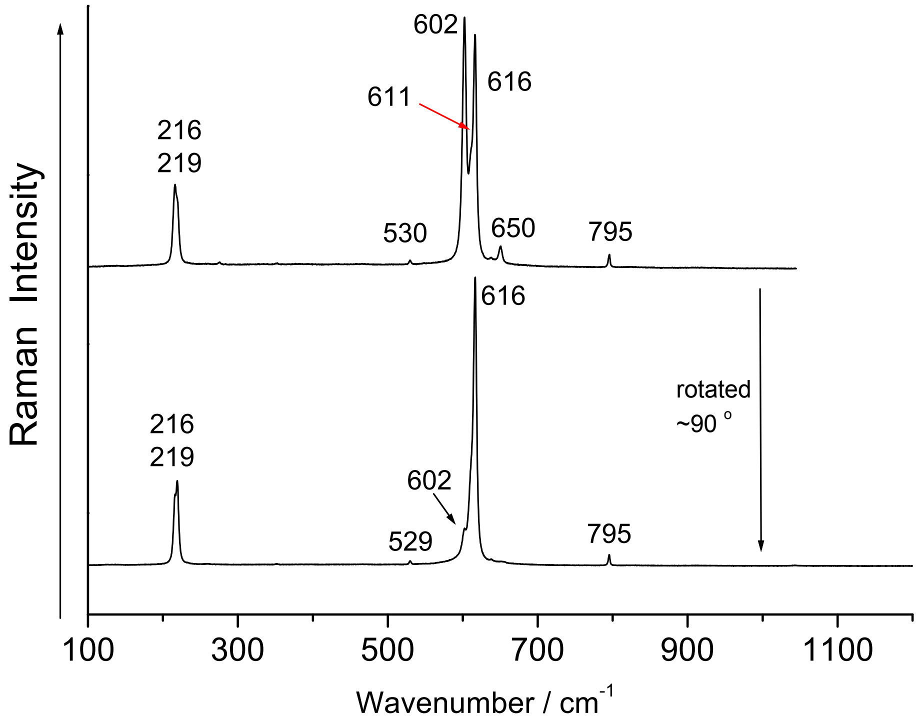

Figure S4. Raman spectra of $Ba(BF_4)(AuF_6)$ recorded on a single crystal.

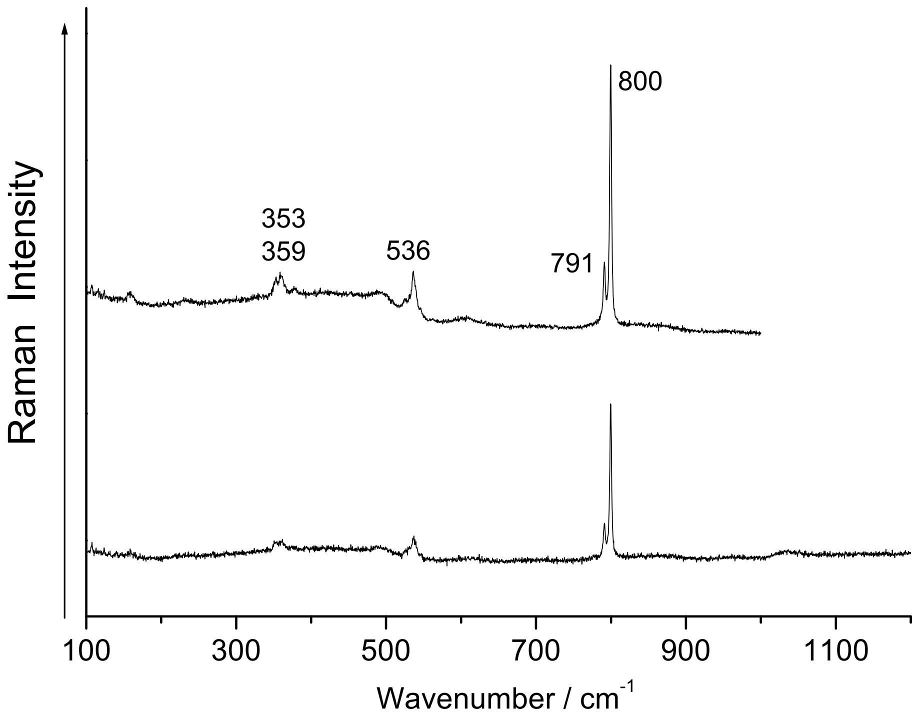

Figure S5. Raman spectra of $[Ca(HF)](BF_4)_2$ recorded on various colorless single crystals.

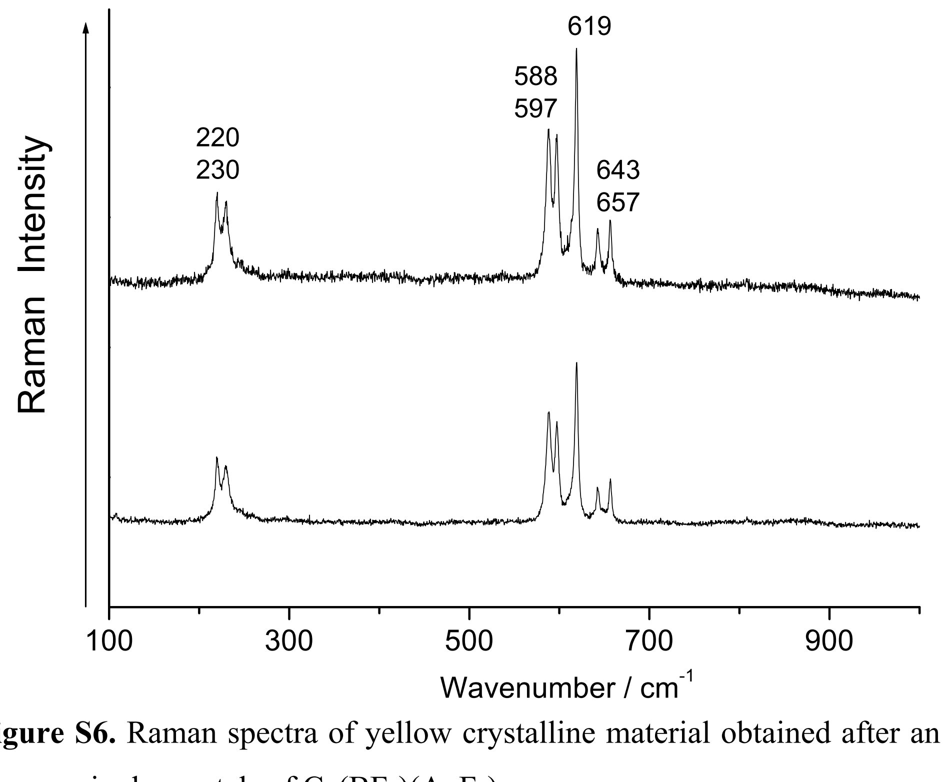

Figure S6. Raman spectra of yellow crystalline material obtained after an experimental attempt to prepare single crystals of $Ca(BF_4)(AuF_6)$.

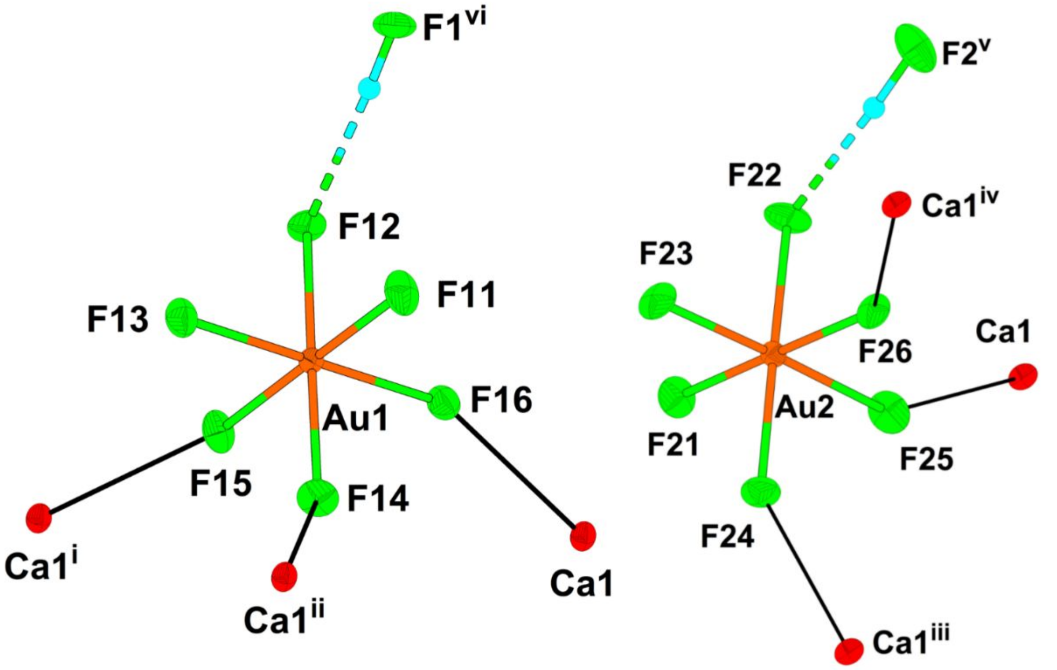

Figure S7. Octahedral coordination of two crystallographically nonequivalent Au atoms (Au1 – left; Au2 – right) in the crystal structure of $[Ca(HF)_2](AuF_6)_2$. The thermal ellipsoids are drawn at the 50 % probability level. The symmetry operations are: (i) 1-x, 1-y, 2-z; (ii) 2-x, 1-y, 2-z; (iii) -1+x, y, z; (iv) 1-x, 1-y, 1-z; (v) 2-x, 1-y, 1-z; (vi) x, 1+y, z.

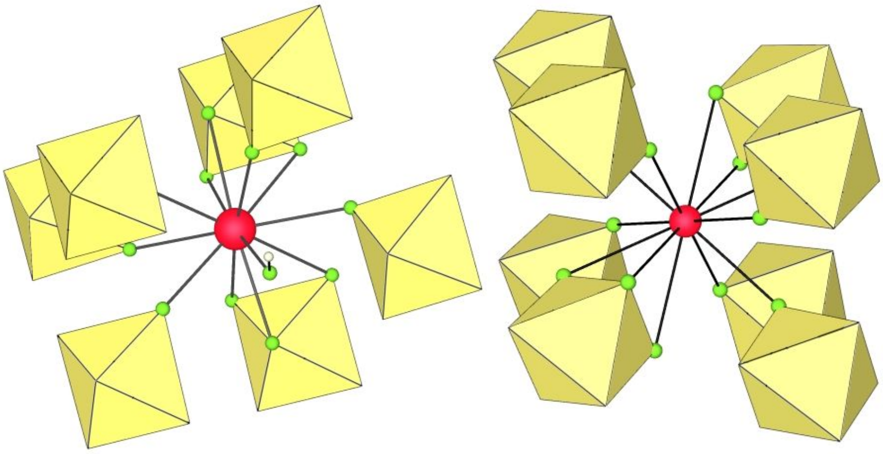

Figure S8. Twelvefold coordination of two crystallographically nonequivalent Ba atoms (Ba1 - left; Ba2 – right) in the crystal structure of $Ba[Ba(HF)]_6(AuF_6)_{14}$ (yellow octahedra: AuF_6; red circles: Ba; green circles: F; small colorless circles: H).

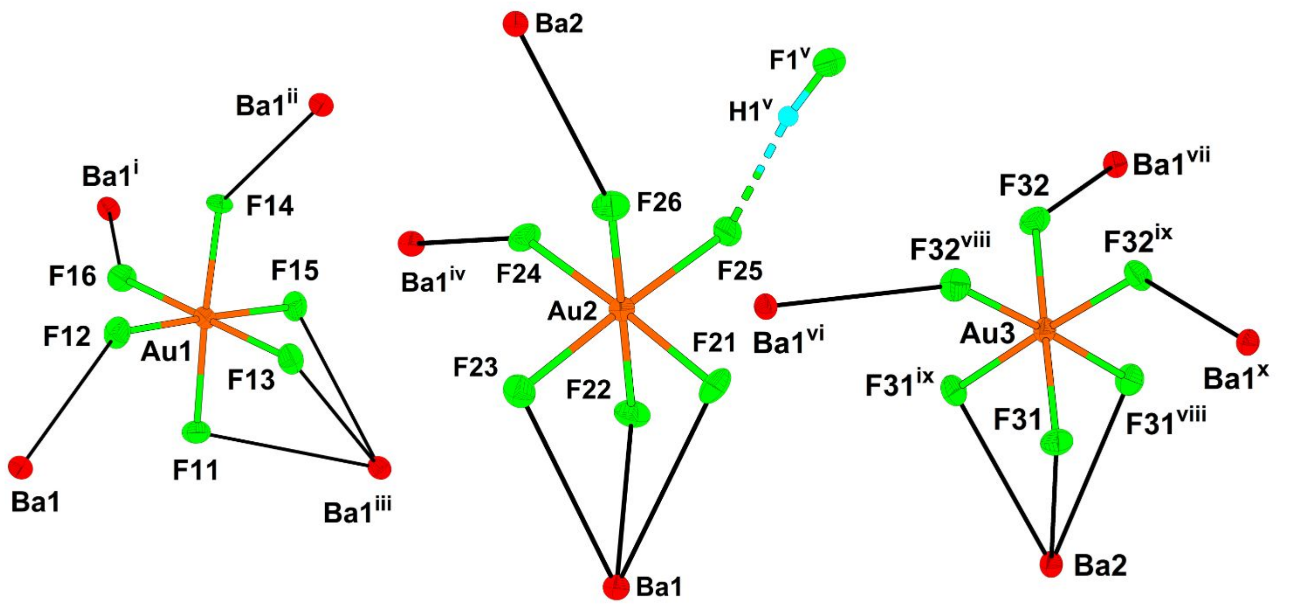

Figure S9. Octahedral coordination of three crystallographically nonequivalent Au atoms (Au1 - left; Au2 - center; Au3 - right) in the crystal structure of $Ba[Ba(HF)]_6(AuF_6)_{14}$. The thermal ellipsoids are drawn at the 50 % probability level. The symmetry operations are: (i) 2/3+x-y, 1/3+x, 4/3-z; (ii) 5/3-x, 4/3-y, 4/3-z; (iii) 4/3-y, 2/3+x-y, -1/3+z; (iv) 1-x, 1-y, 1-z; (v) y, 1-x+y, 1-z; (vi) 2/3-x+y, 4/3-x, 1/3+z; (vii) 2/3-y, 1/3+x-y, 1/3+z; (viii) 1-y, 1+x-y, z; (ix) –x+y, 1-x, z; (x) -1/3+x, 1/3+y, 1/3+z.

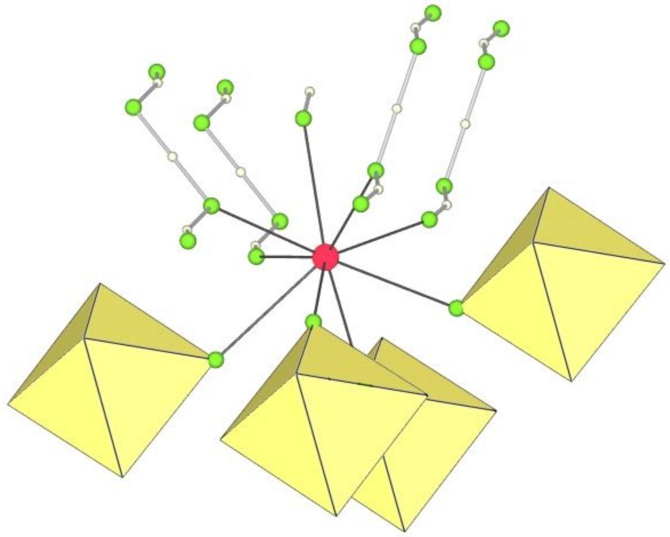

Figure S10. Ninefold coordination of Sr atoms in the crystal structure of $[Sr(HF](H_3F_4)(AuF_6)$ (yellow octahedra: AuF_6; red circles: Sr; green circles: F; small colorless circles: H).

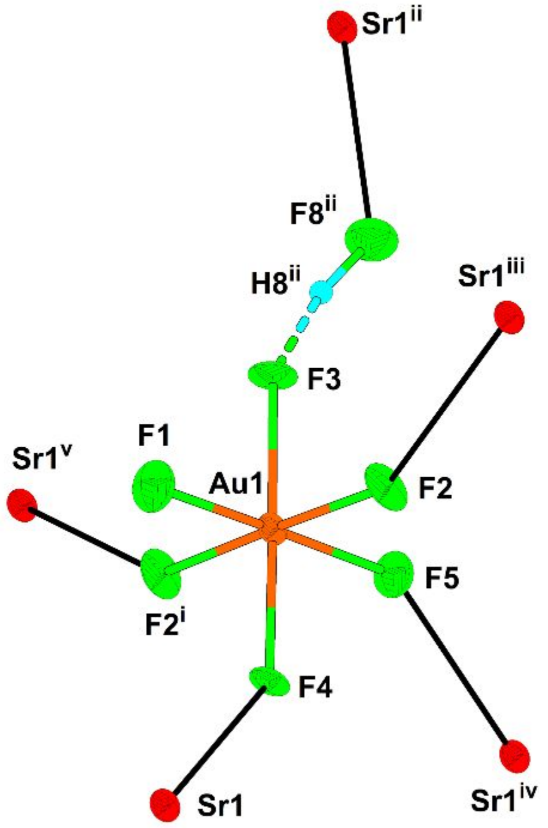

Figure S11. Octahedral coordination of the Au atom in the crystal structure of $[Sr(HF)](H_3F_4)(AuF_6)$. The thermal ellipsoids are drawn at the 70 % probability level. The symmetry operations are: (i) x, ½-y, z; (ii) 1+x, y, -1+z; (iii) 1-x, 1-y, 1-z; (iv) 1+x, y, z; (v) 1-x, -1/2+y, 1-z.

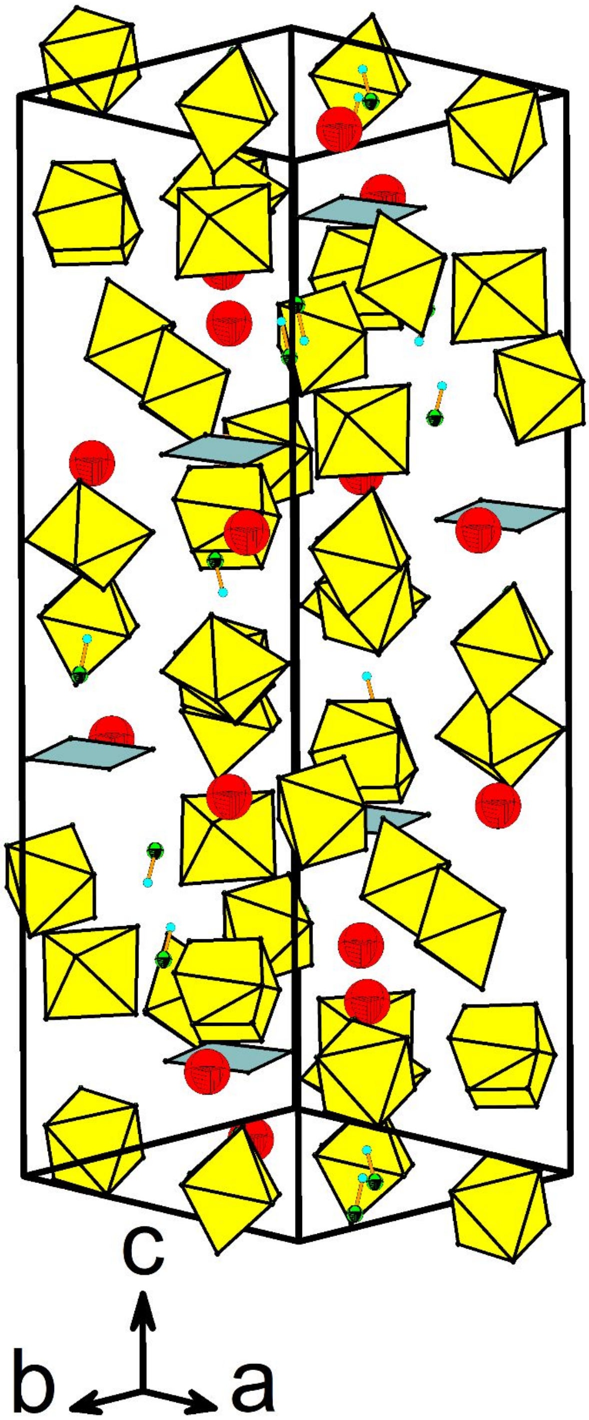

Figure S12. Arrangement of Ba^{2+} cations, $[AuF_6]^-$ and $[AuF_4]^-$ anions, and HF molecules in the crystal structure of $[Ba(HF)]_4(AuF_4)(AuF_6)_7$. For clarity, Ba–F bonds are not shown (yellow octahedra: AuF_6; blue plaques: AuF_4; red circles: Ba; green circles: F; small blue circles: H). The unit cell is also shown. Only one position is shown for each disordered F atom. Unit cell is also shown.

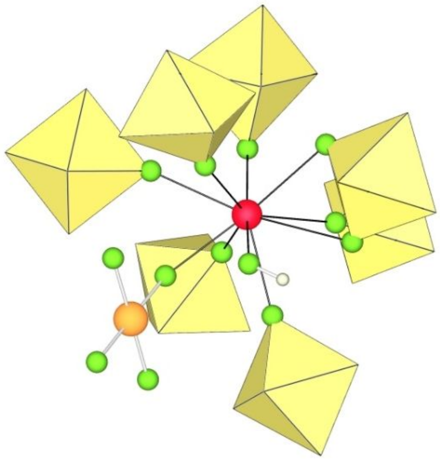

Figure S13. Tenfold coordination of the Ba atom in the crystal structure of $[Ba(HF)]_4(AuF_4)(AuF_6)_7$ (yellow octahedra: AuF_6; orange circles: Au; red circles: Ba; green circles: F; small colorless circles: H). Only one position is shown for each disordered F atom.

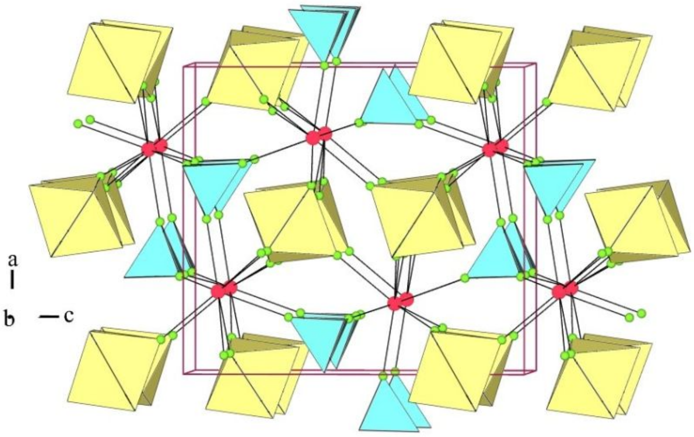

Figure S14. Packing of Sr^{2+} cations, $[AuF_6]^-$ and $[BF_4]^-$ anions in the crystal structure of orthorhombic β-$Sr(BF_4)(AuF_6)$. Only one position is shown for each disordered F atom (yellow octahedra: AuF_6; blue tetrahedra: BF_4; red circles: Sr; green circles: F).

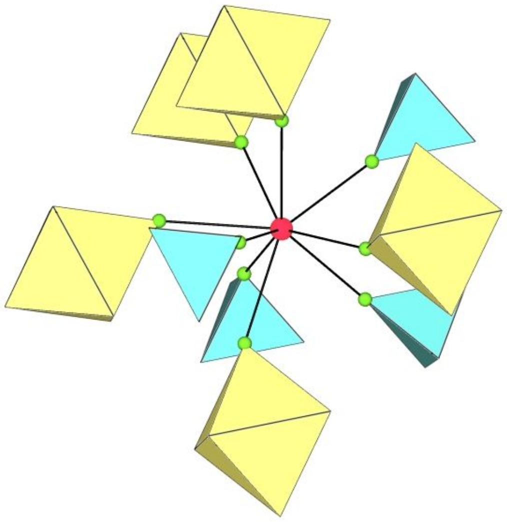

Figure S15. Ninefold coordination of the Sr atom in the crystal structure of monoclinic α-$Sr(BF_4)(AuF_6)$. (yellow octahedra: AuF_6; blue tetrahedra: BF_4; red circles: Sr; green circles: F).

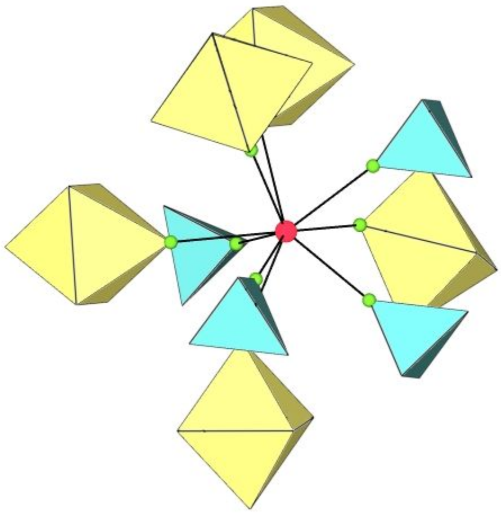

Figure S16. Ninefold coordination of the Sr atom in the crystal structure of orthorhombic β-$Sr(BF_4)(AuF_6)$. Only one position is shown for each disordered F atom (yellow octahedra: AuF_6; blue tetrahedra: BF_4; red circles: Sr; green circles: F).

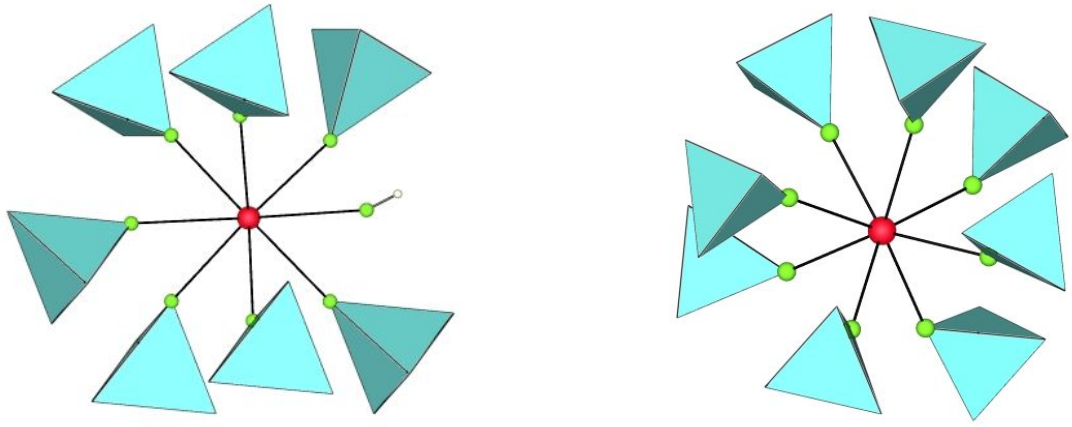

Figure S17. Eightfold coordination of the Ca atom in the crystal structures of $[Ca(HF)](BF_4)_2$ (left) and $Ca(BF_4)_2$ (right; blue tetrahedra: BF_4; red circles: Sr; green circles: F; small colorless circles: H). The image of $Ca(BF_4)_2$ was created using the crystallographic data from the CIF-file.[20]

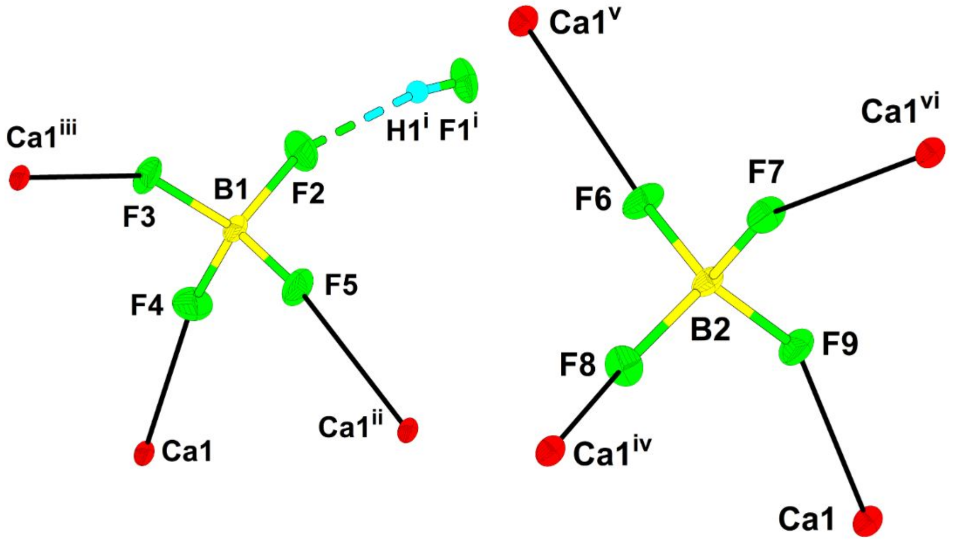

Figure S18. Interactions of two crystallographically independent BF_4 units (B1 - left; B2- right) with Ca^{2+} cations and HF molecule in the crystal structure of $[Ca(HF)](BF_4)_2$. The thermal ellipsoids are drawn at the 50 % probability level. The symmetry operations are: (i) -1+x, -1+y, z: (ii) -1+x, y, z; (iii) 1-x, 1-y, 1-z; (iv) 2-x, 1-y, -z; (v) x, 1-y, z; (vi) 1-x, 1-y, -z.

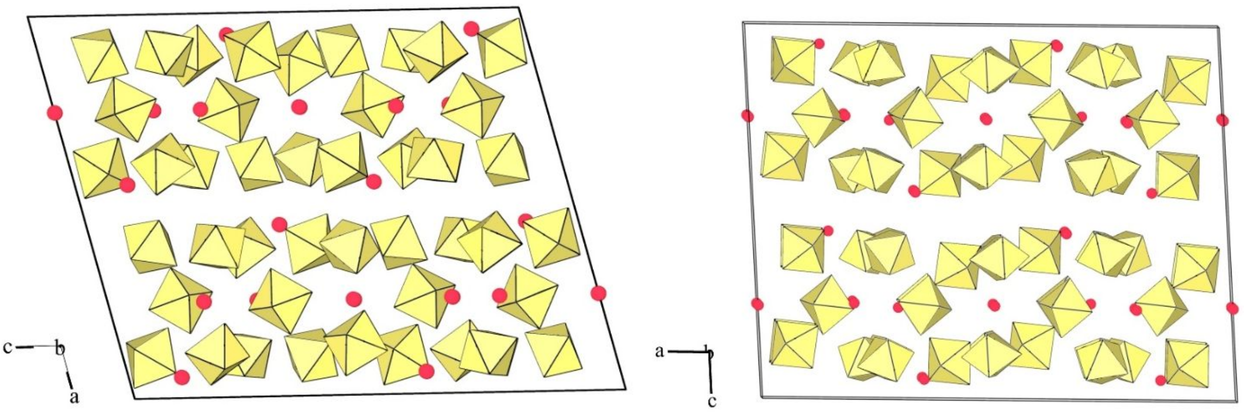

Figure S19. Packing of Sr^{2+} cations and $[AuF_6]^-$ anions in $[O_2]_2[Sr(HF)]_5[AuF_6]_{12}\cdot HF$ (left) and packing of Sr^{2+} cations and $[AsF_6]^-$ anions in $CoSr_5(AsF_6)_{12}\cdot 8HF$ (right); $[XF_6]^-$ (X = Au, As): yellow octahedra; red circles: Sr.

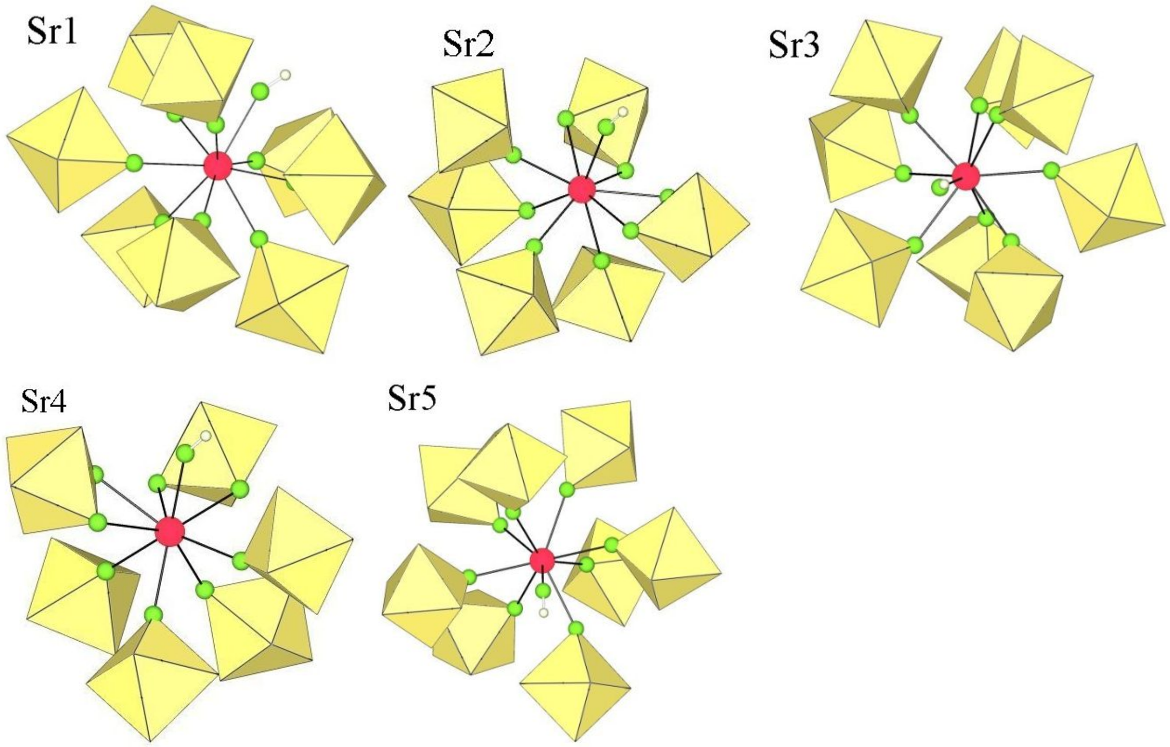

Figure S20. Coordination of five crystallographically unique Sr atoms in the crystal structure of $[O_2]_2[Sr(HF)]_5[AuF_6]_{12}\cdot HF$ (yellow octahedra: AuF_6; red circles: Sr; green circles: F; small colorless circles: H).

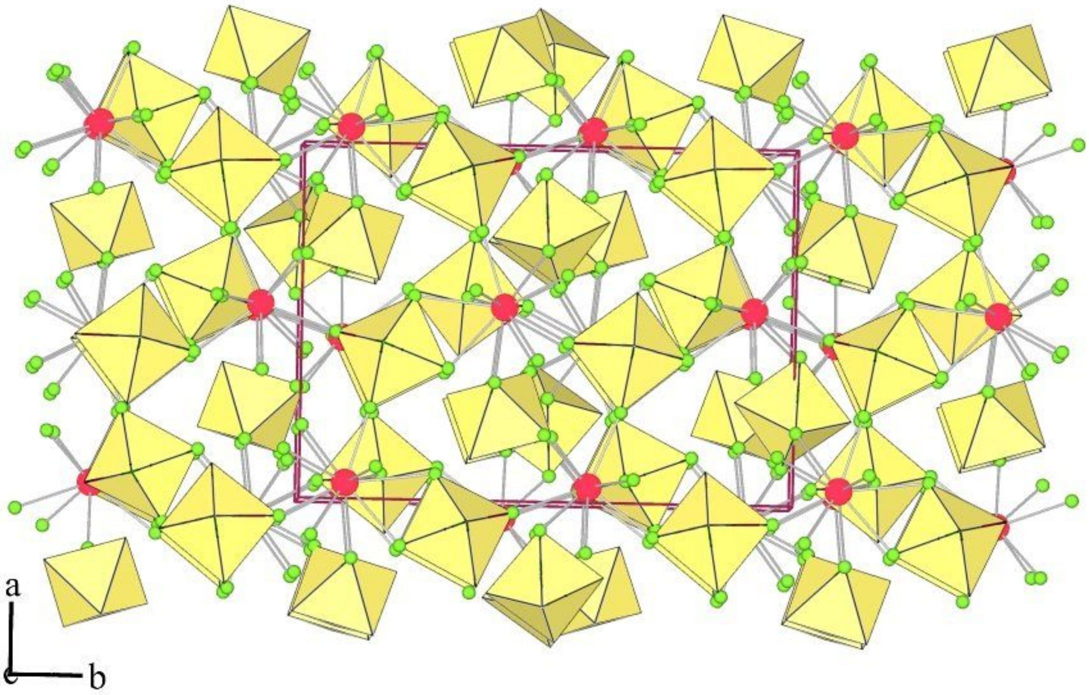

Figure S21. Packing of Sr^{2+} cations, $[AuF_6]^-$ anions and HF molecules in $Sr(HF)(AuF_6)_2$ (H atoms were not located). The unit cell is also shown (orthorhombic; a = 11.1363(3) Å, b = 15.4605(4) Å, c = 10.8490(3) Å at 100 K).

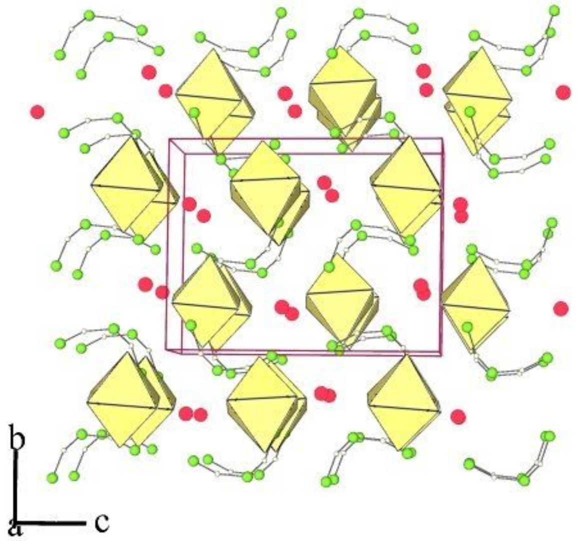

Figure S22. Packing of Sr^{2+} cations and $[H_2F_3]^-$ and $[AuF_6]^-$ anions in $Sr(H_2F_3)(AuF_6)$. The unit cell is also shown (monoclinic, a = 7.3730(13) Å, b = 8.4492(8) Å, c = 11.1099(15) Å; β = 99.276(14) ^{o} at 150 K).

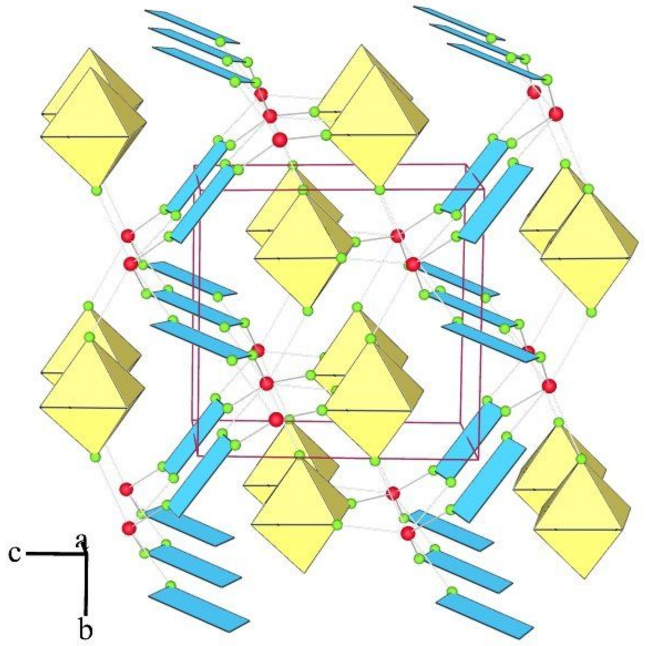

Figure S23. Packing of Ca^{2+} cations and $[AuF_4]^-$ and $[AuF_6]^-$ anions in $Ca(AuF_4)(AuF_6)$. The unit cell is also shown (triclinic, *a* = 5.5175(3) Å, *b* = 8.1945(6) Å, *c* = 8.7795(8) Å, α = 87.001(7) °, β = 72.550(7) °, γ = 89.858(5) ° at 293 K).

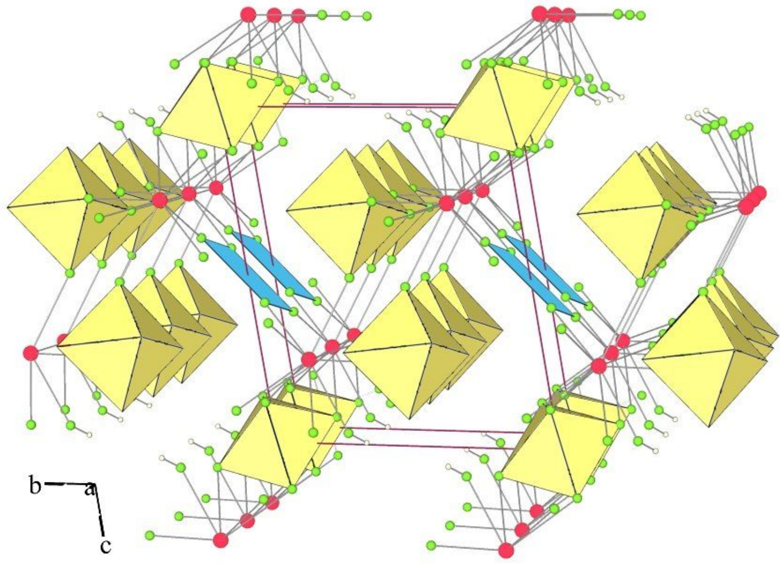

Figure S24. Packing of Sr^{2+} cations, $[AuF_4]^-$ and $[AuF_6]^-$ anions and HF molecules in $[Sr(HF)]_2(AuF_6)_3(AuF_4)$. The unit cell is also shown (triclinic, *a* = 5.6215(6) Å, *b* = 8.7306(7) Å, *c* = 9.9790(8) Å, *α* = 97.522(7) °, *β* = 95.002(8) °, *γ* = 104.747(9) ° at 150 K).

References

[1] Leary, K.; Zalkin, A.; Bartlett, N. Crystal structure of $Xe_2F_{11}^+AuF_6^-$ and the Raman spectrum of $Xe_2F_{11}^+$. *Inorg. Chem.* **1974**, *13*, 775–779.

[2] Sokolov, V. B.; Prusakov, V. N.; Rižkov, A. V.; Drobiševsky, Yu. V.; Horošev, S. S., Syntheses and some properties of gold pentafluoride. *Doklady Akad. Nauk SSSR* **1976**, *229*, 884–887 (in Russian).

[3] Holloway, J. H.; Schrobilgen, G. J., Krypton fluoride chemistry: a route to AuF_5, $KrF^+AuF_6^-$, $Xe_2F_3^+AuF_6^-$, and $NO^+AuF_6^-$: the KrF^+-$XeOF_4$ system. *J. Chem. Soc. Chem. Commun*. **1975**, 623–624.

[4] Adams, C. J.; Bartlett, N., Tautomerism in xenon hexafluoride: an investigation of xenon hexafluoride and its complexes by Raman spectroscopy. *Israel J. Chem.* **1978**, *17*, 114–125.

[5] Lehmann, J.F.; Schrobilgen, G.J., Structural and vibrational characterization of [KrF][AuF_6] and α-[O_2][AuF_6] using single crystal X-ray diffraction, Raman spectroscopy and electron structure calculations. *J. Fluorine Chem.* **2003**, *119*, 109–124.

[6] Nabiev, Sh. Sh., Vibrational spectroscopy of complex compounds of gold pentafluoride. *Russ. Chem. Bull.* **1999**, *48*, 711–717.

[7] Nabiev, Sh. Sh., Vibrational spectra of AuF_5 complexes with nitrogen fluorides and oxofluorides. *Russ. Chem. Bull.* **2012**, *61*, 497–505.

[8] Sokolov, V. B.; Tsinoev, V. G.; Ryzhkov, A. V., Mössbauer effect and the vibrational spectra of AuF_5 and $KrF_2{\cdot}AuF_5$. *Theor. Exp. Chem.* **1980**, 16, 270–272.

[9] Bartlett, N.; Leary, K., Quinquevalent gold salts. *Rev. Chim. Miner.* **1976**, *13*, 82–97.

[10] Yeh, S. M.; Bartlett, N., On the preparation of ReF_6^+ salts: evidence for mixed ion-molecule salts of formula $ReF_6^+ReF_7MF_6^-MF_5$ (M = Sb, Au). *Rev. Chim. Miner.* **1986**, *23*, 676–689.

[11] Shen, C.; Žemva, B.; Lucier, G. M.; Graudejus, O.; Allman, J. A.; Bartlett, N., Disproportionation of Ag(II) to Ag(I) and Ag(III) in fluoride systems and syntheses and structures of $(AgF^+)_2AgF_4^-MF_6^-$ salts (M = As, Sb, Pt, Au, Ru). *Inorg. Chem.* **1999**, *38*, 4570–4577.

[12] Graudejus, O.; Elder, S. H.; Lucier, G. M.; Shen, C.; Bartlett, N. Room temperature syntheses of AuF_6^- and PtF_6^- salts, $Ag^+AuF_6^-$, $Ag^{2+}PtF_6^-$, and $Ag^{2+}PdF_6^-$, and an estimate for E(MF_6^-) [M = Pt, Pd]. *Inorg. Chem.* **1999**, *38*, 2503–2509.

[13] Graudejus, O.; Wilkinson, A. P.; Chacón, L. C.; Bartlett, N., M–F interatomic distances and effective volumes of second and third transition series MF_6^- and MF_6^{2-} anions. *Inorg. Chem.* **2000**, *39*, 2794–2800.

[14] Kiselev, Yu. M.; Popov, A, I.; Sokolov, V. B.; Spirin, S. N., X-ray diffraction study of hexafluoroaurates(V) of alkali metals. *Zh. Neorg. Khim*. **1989**, *34*, 434–437 (in Russian).
[15] Mazej, Z.; Hagiwara, R. Hexafluoro-, heptafluoro-, and octafluoro-salts, and $[M_nF_{5n+1}]^-$ (n = 2, 3, 4) polyfluorometallates of singly charged metal cations, Li^+ - Cs^+, Cu^+, Ag^+, In^+ and Tl^+. *J. Fluorine Chem.* **2007**, *128*, 423–437.
[16] Mazej, Z.; Goreshnik, E., X-ray single crystal structures of $Cd(AuF_6)_2$, $Mg(HF)AuF_4AuF_6$ and $KAuF_6$ and vibrational spectra of $Cd(AuF_6)_2$ and $KAuF_6$. *Solid State Sciences* **2006**, *8*, 671–677.
[17] Popov, A, I.; Val'kovskii, M. D.; Kiselev, Yu. M.; Tchumaevsky, N. A.; Sokolov, V. B.; Spirin, S. N., Structures of fluoroaurates(V) of alkaline earth elements. *Zh. Neorg. Khim*. **1990**, *35*, 1970–1977 (in Russian).
[18] Mazej, Z., Recent achievements in the synthesis and characterization of metal hexafluoroantimonates and hexafluoroaurates. *J. Fluorine Chemistry* **2004**, *125*, 1723–1733.
[19] Mazej, Z.; Goreshnik, E.; Tavčar, G., X-ray single crystal structures of $Hg(AuF_6)_2$ and $AgFAuF_6$, *J. Fluorine Chemistry* **2011**, *132*, 686–689.
[20] Jordan, T. H.; Dickens, B.; Schroeder, L. W.; Brown, E. E. The crystal structure of $Ca(BF_4)_2$. *Acta Crystallogr. Sec. B* **1975**, *31*, 669–672.
